# Hepatic stellate cell subpopulations in human MASLD-related hepatocellular carcinoma upregulate YAP via geranylgeranyl pyrophosphate

**DOI:** 10.1097/HC9.0000000000001015

**Published:** 2026-08-14

**Authors:** Courtney L. Labrecque, Arthur Ko, Bo Qiao, Calvin Pan, Renu Nandakumar, Kushan Chowdhury, Namrata Venkatesan, Xiaotang Du, Bishuang Cai, Matteo Pellegrini, Joseph R. Pisegna, Samuel W. French, Vatche Agopian, Enrique Rozengurt, Rajat Singh, Jihane N. Benhammou

**Affiliations:** 1 Veterans Affairs Greater Los Angeles Healthcare System, Los Angeles, California, USA; 2 David Geffen School of Medicine, University of California Los Angeles, Los Angeles, California, USA; 3 Comprehensive Liver Research Center, University of California, Los Angeles, California, USA; 4 Center for Precision Medicine and Genomic Research, Children’s National Research Institute, Washington, District of Columbia, USA; 5 Columbia University Biomarkers Core Laboratory, New York, New York, USA; 6 Vatche and Tamar Manoukian Division of Digestive Diseases, University of California Los Angeles, Los Angeles, California, USA; 7 Department of Pathology, University of California Los Angeles, Los Angeles, California, USA; 8 Department of Human Genetics, University of California Los Angeles, Los Angeles, California, USA; 9 Department of Surgery, University of California Los Angeles, Los Angeles, California, USA; 10 Jonsson Comprehensive Cancer Center, University of California Los Angeles, Los Angeles, California, USA

**Keywords:** geranylgeranyl pyrophosphate, hepatocellular carcinoma, Rho GTPases, spatial imaging, single-nucleus RNA sequencing, statins, yes-associated protein

## Abstract

**Background:**

Hepatic stellate cells (HSCs) drive cirrhosis and hepatocellular carcinoma (HCC). Statin use decreases cirrhosis and HCC through unclear mechanisms. We aimed to uncover statin-responsive pathways preventing activation of HSC subpopulations in human MASLD-HCC.

**Methods:**

We used single-nucleus RNA-sequencing and spatial imaging on matched human MASLD-HCC samples in cirrhotic and non-cirrhotic livers, along with RNA-sequencing of human primary HSCs to identify Yes-Associated Protein (YAP) as a statin-responsive pathway, which was assessed for cellular localization and function, that is, downstream gene expression after statin exposure. We used pharmacologic inhibitors, gene silencing, pharmacological repletion, and direct quantification by UPLC-MS/MS to interrogate the mevalonate pathway as a statin-responsive YAP regulator.

**Results:**

We identified an HCC-HSC subcluster enriched in activated and deactivated marker genes. Spatial resolution of each cell type revealed that myofibroblast-like HSC subpopulations and YAP-effector genes colocalized in the peritumoral pseudocapsule. In fact, although Rho GTPases were differentially expressed across most cell types, YAP-effector genes were largely upregulated in HCC-HSCs by single-nucleus RNA sequencing. Bulk RNA-sequencing of statin-treated activated human primary HSCs showed decreased expression of YAP-related genes and downregulation of Rho GTPase pathways in response to statins. Statins reduced GGPP levels in LX2 HSCs, determined by direct measurement of intracellular GGPP, which was associated with cytoskeletal restructuring, YAP cytosolic retention, and reduced YAP-effector gene expression. Exogenous GGPP repletion reversed these effects. *GGPP synthase 1* knockdown and direct Rho inhibition recapitulated the cellular responses of YAP following statin exposure.

**Conclusions:**

Upregulation of YAP-effector genes was largely restricted to HCC-HSCs. Our mechanistic studies support that statins lower GGPP, reduce Rho GTPases prenylation, cause YAP cytosolic retention, and decrease YAP nuclear activity. This study expands on the chemoprotective mechanisms of statins in HCC.

## Graphical abstract

**Figure:**
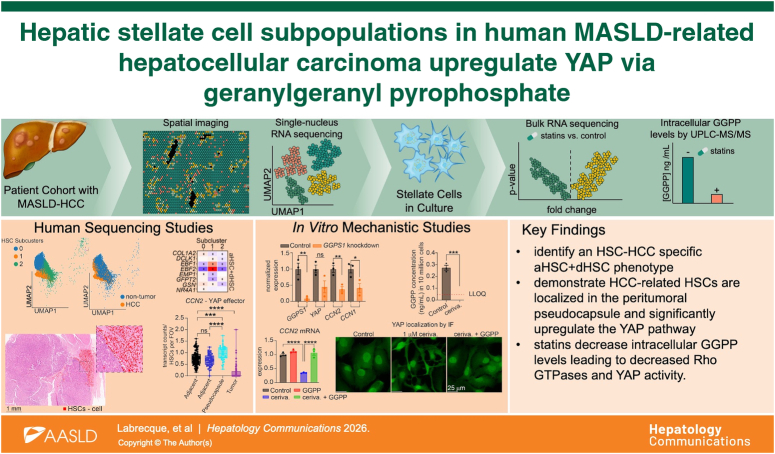

## INTRODUCTION

Metabolic dysfunction–associated steatotic liver disease (MASLD) is the most common cause of chronic liver disease, affecting a third of the world population.^1^ This has led to a surge in deaths and liver transplantations attributed to MASLD-related hepatocellular carcinoma (HCC) and its complications,^2^^,^^3^ making HCC the third leading cause of cancer-related deaths worldwide.^4^

While predominantly occurring in a background of cirrhosis,^5^ MASLD-HCC arises in the absence of advanced fibrosis in 25%–30% of cases,^6^^,^^7^ suggesting distinct carcinogenesis mechanisms. Efforts to curb the rise of HCC have focused on surveillance-based, biomarker-based, and chemoprevention-based methods, including statins. Statin chemoprevention is being investigated only in high-risk patients with cirrhosis,^8^^,^^9^ leaving how to prevent MASLD-HCC in its absence a clinical dilemma.

Central to the development of hepatic fibrosis, cirrhosis, and HCC is the hepatic stellate cell (HSC). Chronic liver injury triggers the net accumulation of extracellular matrix (ECM) and hepatic fibrosis.^10^ In normal livers, quiescent HSCs, defined by their cytoplasmic vitamin A-storing lipid droplets, maintain ECM homeostasis, metabolic regulation, and vasoregulation.^10^^–^^12^ During injury, HSCs activate and transdifferentiate into proliferative lipid droplet-depleted myofibroblast-like cells, which express α-smooth muscle actin (*α-SMA*) and ECM proteins, including collagen and fibronectin, resulting in cirrhosis if injury persists.^11^^,^^13^^,^^14^ However, HSCs in HCC remain functionally dynamic with quiescent HSC subpopulations serving to protect against HCC through cytokine production, while activated HSCs increase hepatocyte proliferation^5^ and modify the HCC microenvironment.^15^ Subpopulations of HSCs also exist in human diseased livers and preclinical models, pointing to their heterogeneity in pathophysiology within the MASLD spectrum.^5^^,^^16^^,^^17^

To better understand HSCs in the MASLD-HCC spectrum using an unbiased approach, we generated one of the largest MASLD-HCC cohorts to identify novel disease pathways and performed single-nucleus RNA sequencing (snRNAseq) and spatial imaging to define HSC subpopulations in cirrhotic and non-cirrhotic HCCs, which revealed a selective upregulation of Yes-associated protein (YAP)-effector genes in fibrosis-related and HCC-related HSCs. While examining statins as chemopreventive agents, we revealed that the mevalonate pathway metabolite geranylgeranyl pyrophosphate (GGPP) stimulates YAP signaling to regulate HSC activation in the context of MASLD-HCC.

## METHODS

### Study design

The study was approved by appropriate ethics committee and the Institutional Review Boards of the University of California, Los Angeles (IRB #20-001319). All participant informed consent was waived given the retrospective nature of the study as all original samples were approved for biobanking and future studies. We conducted a retrospective study of patients undergoing liver resection or transplantation for HCC (including 1 patient with normal tissue from a hepatic adenoma resection) at the Dumont-UCLA Liver Cancer Center. All patients with a diagnosis of “MASLD,” “MASH,” or “cryptogenic” liver disease were included and reviewed by clinical criteria and histopathology (Jihane N. Benhammou and Samuel W. French). All patients had features of steatosis, inflammation (with/without steatohepatitis), and ballooning as per the NAFLD Activity Score.^18^ If patients had any locoregional therapy before transplantation, only non-necrotic tissue was selected for sequencing. All research was conducted in accordance with the Declaration of Helsinki and Istanbul.

### Chemicals and reagents

Cerivastatin, atorvastatin, and dimethylsulfoxide (DMSO) were obtained from Sigma-Aldrich (St. Louis, MO). GGPP was obtained from Cayman Chemical (63330, Ann Arbor, MI); BAY-593 was obtained from MedChemExpress (HY-161573, Monmouth Junction, NJ); and Rho inhibitor I was obtained from Cytoskeleton, Inc. (no. CT04, Denver, CO). CosMx reagents, including sulfo-NHS-acetate, deionized formamide, and 4x saline-sodium citrate (SSC) buffer, were all purchased from Thermo-Fisher (26777, AM9342, and AM9763, Waltham, MA). For immunohistochemistry (IHC) antibodies , GLUL was purchased from Santa Cruz Biotechnology (#sc-74430, Dallas, TX), and HAMP was purchased from Thermo-Fisher (MA5-32669). Biotinylated antibodies were purchased from Vector Laboratories (goat anti-mouse: BA-9200; goat anti-rabbit: BA-1000, Newark, CA). Sodium citrate antigen retrieval buffer, hematoxylin, and a hydrophobic pen were also from Vector Laboratories (H-3300-250, H-3404-100, and H-4000). A 10% goat serum and ABC peroxidase standard staining kit were purchased from Thermo-Fisher (50062Z and 32020). DAB substrate and limonene mounting media were purchased from Abcam (ab64238 and ab104141, Waltham, MA). For additional antibodies, YAP for IF was obtained from Santa Cruz Biotechnology (sc-101199), YAP and p-YAP^127^ for western blots were purchased from Cell Signaling (14074 and 13008, Danvers, MA), Alexa Fluor 488 (A11001) and Alexa Fluor 546 Phalloidin (A22283) were purchased from Thermo-Fisher, and Hoechst 33258 was purchased from Sigma-Aldrich (94403, St. Louis, MO). All qPCR primers were synthesized by IDT.

Additional Methods are in the Supplemental Material, https://links.lww.com/HC9/C448.

## RESULTS

### HSCs are enriched in cirrhotic versus non-cirrhotic and pre-cirrhotic livers, uninfluenced by tumor status

**FIGURE 1:**
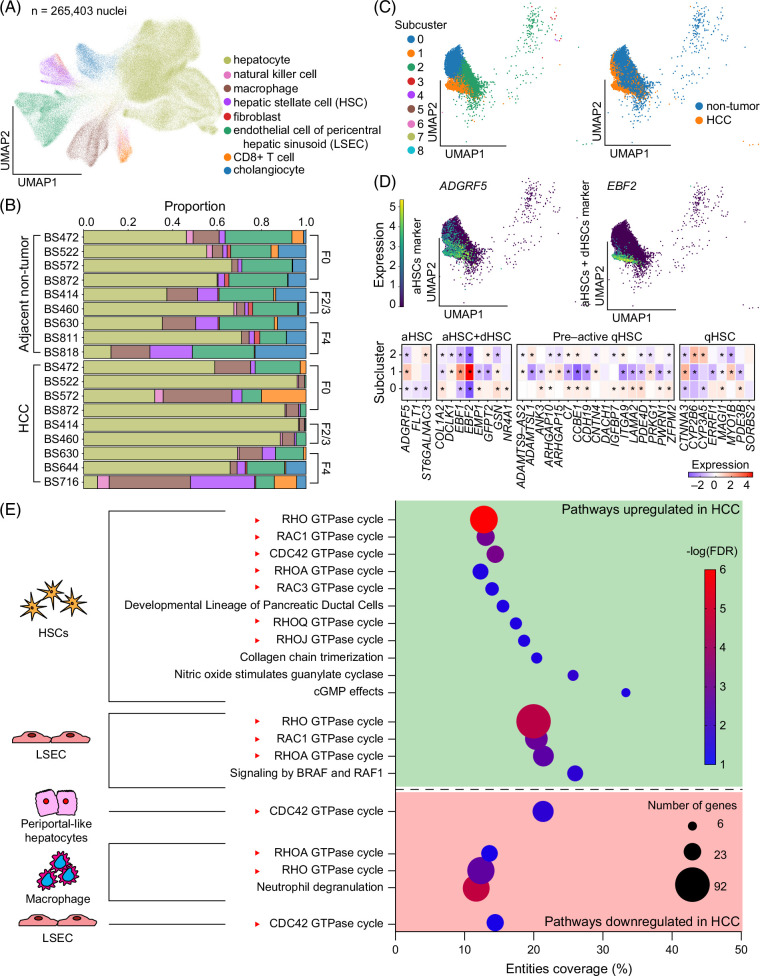
snRNAseq identifies the role of Rho GTPases in HCC tissues. (A) UMAP plot of 8 cell-type clusters from snRNAseq (n=11 donors) in adjacent non-tumor (n=9) and HCC tissues (n=9). (B) Cell-type proportion plot identifies heterogeneity of individual patients. (C) HSCs subclustering revealed 3 main clusters with >20 cells per cluster. (D) Subcluster 1 was found to be predominantly in HCC tissues with a high expression of aHSCs+dHSCs markers. Heatmap showing the top marker genes per cluster based on previously reported HSC marker genes available in our data (n=36 marker genes). *FDR<0.05. (E) Pathway analyses of DEGs by Reactome identify Rho and related GTPases (red arrows) as key pathways in HCC tissues. Abbreviations: aHSCs, activated hepatic stellate cells; DEGs, differentially expressed genes; dHSCs, damaged hepatic stellate cells; FDR, false discovery rate; GTPases, guanosine triphosphatases; HCC, hepatocellular carcinoma; HSCs, hepatic stellate cells; snRNAseq, single-nucleus RNA sequencing; UMAP, Uniform Manifold Approximation and Projection.

To understand the pathobiology of HSCs in MASLD-HCC, we sought to examine the spectrum of disease with and without cirrhosis. We identified 11 patients with MASLD-HCC, most of whom had histologically moderately differentiated HCCs (n=5) (Table 1) with the presence (n=5 with F4) or absence of cirrhosis (n=4 with F0 and n=2 with F2–F3). Patients with cirrhotic HCCs commonly self-identified as Hispanic (n=4) and were younger (58.8±8.6 y vs. 75.5±3.0 y; *p*=0.0015), with a trend toward having smaller tumors [2.9 cm (IQR 2.2–4.8 cm) vs. 6.0 cm (IQR 2.6–13.3 cm)] when compared with patients with non-cirrhotic or pre-cirrhotic HCCs (Table 1). Copy number variation (CNV) analyses on the subset of samples with available formalin-fixed paraffin-embedded (FFPE) tissues (n=5) confirmed that the tumors were HCCs as evidenced by expected deletions and amplifications of HCC marker genes. To identify cell types in MASLD-HCC and compare gene expression profiles of HSCs based on the presence or absence of cirrhosis, we generated snRNAseq data from 9 adjacent non-tumor and 9 HCC samples from 11 unique patients, one of which was an integrated dataset from our previous study (BS472) (Table 1).^19^ We identified 265,403 nuclei resulting in 8 cell types based on the most highly expressed genes: hepatocytes, which could be further resolved into periportal-like, midzonal-like, and centrilobular-like, cholangiocytes, CD8+ T-cells, endothelial cells of pericentral hepatic sinusoid (LSECs), fibroblasts, HSCs, macrophages, and natural killer cells (NK cells) (Figure 1A). While there was cell-type heterogeneity across all samples, hepatocytes were predominant in all samples besides BS818 adjacent non-tumor and BS716 HCC (Figure 1B and Supplemental Figure S1A, https://links.lww.com/HC9/C448). As expected, cirrhotic samples had higher numbers of HSCs (925 ±690 cells) compared with non-cirrhotic and pre-cirrhotic samples (293 ±303 cells, *p*=0.0103), regardless of tumor status (Supplemental Figures S1B–D, https://links.lww.com/HC9/C448).

**TABLE 1:** Clinical demographics of the MASLD-related HCC cohort

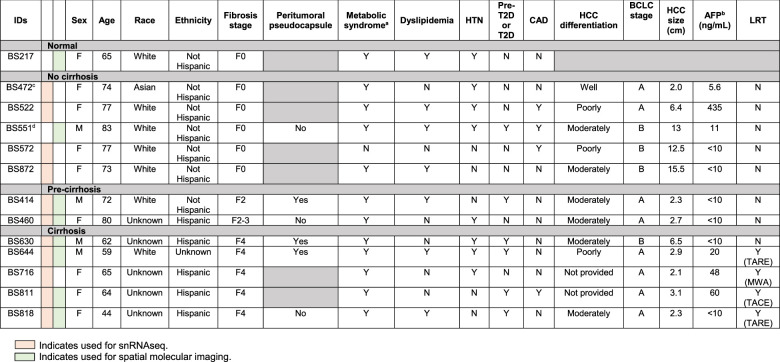

^a^Metabolic syndrome = dyslipidemia or hypertension or type 2 diabetes.

^b^Alphafeto protein (AFP) at the time of diagnosis, LRT, MWA, TACE, TARE (or Y90).

^c^Data integrated from previous snRNAseq (see the Methods section).

^d^BS511-HCC is a core biopsy.

Abbreviations: AFP, alpha-fetoprotein; BCLC, Barcelona Clinic Liver Cancer; CAD, coronary artery disease; F, female; HCC, hepatocellular carcinoma; HTN, hypertension; LRT, locoregional therapy; M, male; MASLD, metabolic dysfunction–associated steatotic liver disease; MWA, microwave ablation; snRNAseq, single-nucleus RNA sequencing; T2D, type 2 diabetes; TACE, transarterial chemoembolization; TARE, transarterial radioembolization; Y90, yttrium-90.

### Heterogeneity of HSC subpopulations in HCC tissue

HSC subpopulations have previously been identified in HCC and adjacent non-tumor tissue with divergent mechanisms.^5^ Given its known heterogeneity, we sought to further characterize HSC subpopulations in our cohort. Subclustering all HSCs revealed 3 main subclusters (subcluster 0, n=3,468 cells; subcluster 1, n=1,757 cells; and subcluster 2, n=3,601 cells) (Figure 1C and Supplemental Figure S2A, https://links.lww.com/HC9/C448). Using validated markers for quiescent (qHSCs) and activated (aHSCs) HSCs,^20^^–^^22^ we identified that subclusters 0, 1, and 2 represented the spectrum of quiescent to activated and deactivated states of HSCs in both tumor and non-tumor adjacent tissue, supporting HSC functional heterogeneity across disease states (Figures 1C, D and Supplemental Figures S2B, C, https://links.lww.com/HC9/C448). Interestingly, subcluster 1 was predominantly present in HCC tissue compared with subclusters 0 and 2 (Figure 1C). To determine the nature and state of HCC-enriched subcluster 1, we conducted differenential expression (DEGs) of subcluster 1 versus all other subclusters (Supplemental Table S1, https://links.lww.com/HC9/C448) and observed that qHSC marker genes, including *THBS1* (β=−1.47, *FDR*=3.85E−19) and *GEM* (β=−1.09, *FDR*=2.96E−21), were markedly downregulated, compared with other subclusters (Supplemental Figures S2B, C, https://links.lww.com/HC9/C448, and Supplemental Table S1, https://links.lww.com/HC9/C448). We also found that subcluster 1 had a mixed expression of fibrosis-related genes, including an increase in *ACTA2* (β=0.63, *FDR*=1.28E−07) and a decrease in *COL1A2* (β=−0.67, *FDR*=9.18E−10), suggesting an activated and deactivated phenotype of HSCs (aHSCs+dHSCs) (Supplemental Figure S2B, https://links.lww.com/HC9/C448, and Supplemental Table S1, https://links.lww.com/HC9/C448). Accordingly, aHSCs+dHSCs marker genes^22^ were significantly increased, including *EBF1* (β=2.53, *FDR*=0.00) and *EBF2* (β=4.65, *FDR*=1.32E−58) (Figure 1D and Supplemental Figure S2B, https://links.lww.com/HC9/C448). Pathway analyses of the DEGs in subcluster 1 versus others showed significant enrichment of genes related to the term “ECM organization” (*FDR*=1.92E−12) (Supplemental Table S1, https://links.lww.com/HC9/C448).

Compared with all other subclusters, subcluster 0 was enriched with known “pre-active qHSC” marker genes, including *PDED4* (β=0.82, *FDR*=4.74E−123), *LAMA2* (β=0.71, *FDR*=2.90E−70), and *ANK3* (β=0.49, *FDR*=4.81E−39) (Figure 1D, Supplemental Figures S2B, C, https://links.lww.com/HC9/C448, and Supplemental Table S1, https://links.lww.com/HC9/C448). We found that all significantly upregulated/downregulated genes were enriched in various signal transduction and Rho GTPase cycle pathways by pathway analyses (Supplemental Table S1, https://links.lww.com/HC9/C448). Subcluster 2 had a more qHSC gene profile compared with all other subclusters, with marker genes *CYP2B6* (β=1.72, *FDR*=6.18E−4) and *CYP3A5* (β=1.34, *FDR*=2.60E−5) exclusively upregulated in the subcluster (Figure 1D, Supplemental Figure S2C, https://links.lww.com/HC9/C448, and Supplemental Table S1, https://links.lww.com/HC9/C448). However, subcluster 2 did not have any significantly enriched pathways of its DEGs when compared with other subclusters.

### Rho GTPases and Yes-associated protein are upregulated in HSC-HCCs

To identify differences in cell types based on tumor and cirrhosis status, we analyzed DEGs and conducted pathway analyses in the most abundant cell types (Supplemental Table S2, https://links.lww.com/HC9/C448). Perturbations in gene expression in HCCs were compared with gene expression in adjacent non-tumor regions from the same individuals (paired samples, n=7), while identification of fibrosis-specific gene changes was done by comparing adjacent non-tumor cirrhotic regions (n=3 with F4) to adjacent non-tumor regions without cirrhosis (n=4 with F0). When examining HCC tissues, Rho GTPases were remarkably upregulated in HSCs (*FDR*<0.05) (Figure 1E and Supplemental Table S3, https://links.lww.com/HC9/C448). Conversely, when comparing cirrhosis to non-cirrhosis livers in tumor-free regions, Rho and related GTPases were significantly downregulated exclusively in HSCs (*FDR*<0.05) (Figure 1D, Supplemental Figure S3, https://links.lww.com/HC9/C448, and Supplemental Table S3, https://links.lww.com/HC9/C448). This directly inverse relationship between Rho pathways in HSCs in cirrhosis and HCC was not seen in any other cell types and suggests a potential role of Rho GTPases in HCC-HSCs influencing HCC development and its microenvironment (Figure 1E, Supplemental Figure S3, https://links.lww.com/HC9/C448, and Supplemental Table S3, https://links.lww.com/HC9/C448).

Due to the link between Rho GTPases and YAP activity in HCC,^23^^,^^24^ and because YAP is known to regulate HSC activation,^25^ we hypothesized that the upregulation of Rho activates YAP-effector genes in HCC-HSCs. Interestingly, although we did not see significant differences in YAP-target gene expression broadly across tumor vs. adjacent non-tumor regions (Supplemental Table S2, https://links.lww.com/HC9/C448, and Supplemental Figure S4A, https://links.lww.com/HC9/C448), when separated into cell-type-specific changes, we found that 2 defined YAP-effector genes, *CCN1* (β=0.98, *FDR*=2.61E−06) and *CCN2* (β=0.66, *FDR*=6.2E−04), were both significantly upregulated only in the HCC-HSCs, when compared with HSCs from adjacent non-tumor controls (Supplemental Table S2, https://links.lww.com/HC9/C448, and Supplemental Figure S4B, https://links.lww.com/HC9/C448). Consistently, other validated YAP-target genes^26^ were also upregulated in HCC-HSCs, while *CCN1,* but not *CCN2*, was upregulated in HSCs of cirrhotic samples, when compared with HSCs from non-cirrhotic samples (Supplemental Table S2, https://links.lww.com/HC9/C448, and Supplemental Figure S4B, https://links.lww.com/HC9/C448). Taken together, these data demonstrate Rho GTPases and YAP activity in HSCs in the cirrhosis-HCC spectrum.

### Spatial imaging reveals the distribution of HSCs in the peritumoral pseudocapsule

**FIGURE 2:**
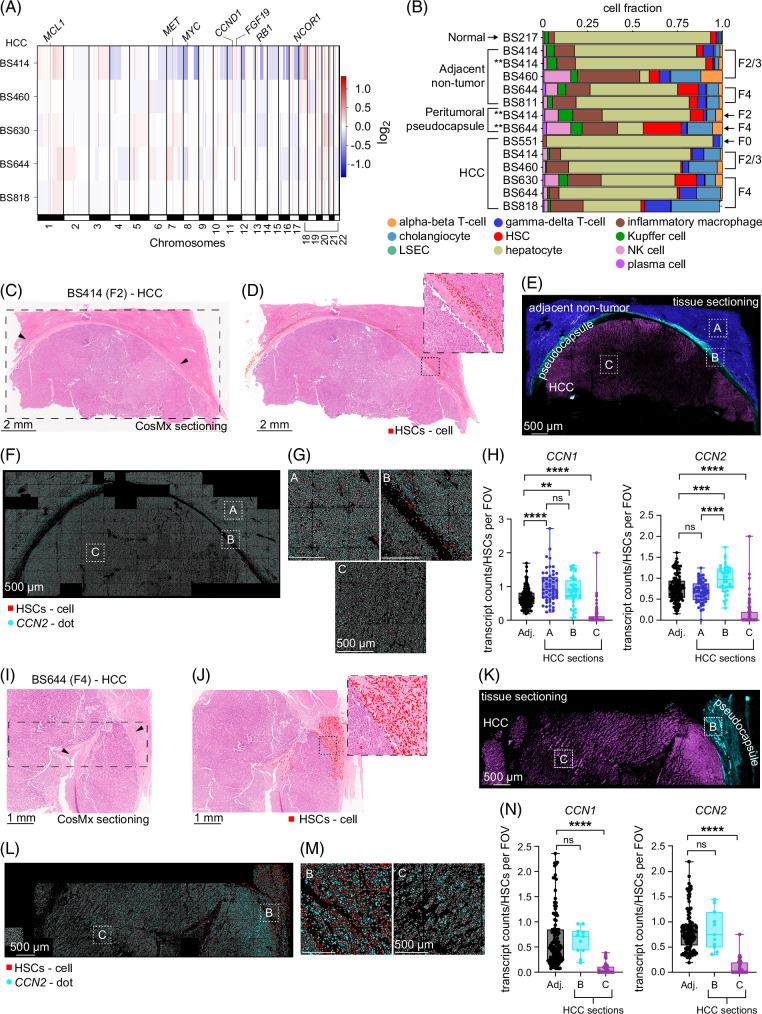
HSCs are enriched in the peritumoral pseudocapsule of HCCs. (A) Copy number variation analysis of the whole genome of 5 patients with HCC confirms the presence of known amplifications/deletions of select HCC marker genes. (B) Cell-type proportions of spatial imaging samples (n=8 patients: n=1 normal, n=4 adjacent, n=6 HCCs). **Denotes sectioning of HCC tissue. (C) H&E of BS414-HCC shows a peritumoral pseudocapsule (arrows) separating the tumor from the adjacent tissue. (D) This peritumoral pseudocapsule has a high population of HSCs, shown by an overlay of H&E with spatial imaging. (E) Sectioning of the HCC section into: adjacent (A), peritumoral pseudocapsule (B), and HCC (C) for comparisons between the tissue sections. (F, G) Spatial imaging of *CCN2* (cyan) and HSCs (red) in BS414-HCC shows not only a higher population of HSCs in the pseudocapsule, but also a higher expression of the YAP-effector genes. (H) Quantification of *CCN1* and *CCN2* expression levels in HSCs by segmentation shows that these genes are upregulated in the pseudocapsule (segment B) relative to the adjacent tissue. FOVs n = 40–124, **p=0.0055, ***p=0.0004, and ****p<0.0001 by Mann–Whitney *U* test. (I–N) Represent the cirrhotic (BS644) sample. FOVs n=11–104 and ****p<0.0001 by Mann–Whitney *U* test. Abbreviations: H&E, hematoxylin and eosin; CCN1, cellular communication factor 1; CCN2, cellular communication factor 2; FOVs, fields of view; HCC, hepatocellular carcinoma; HSCs, hepatic stellate cells; YAP, Yes-associated protein.

The zonal distribution of parenchymal and non-parenchymal cells across the liver lobule is central to their function. To characterize how HSCs and other cell types are spatially distributed in MASLD-HCC at the single-cell level, we assessed zone-specific gene expression patterns in these cell types using the *Nanostring* CosMx spatial imager. We imaged 11 samples from 8 patients: 1 normal, 4 adjacent non-tumors (n=2 with F2–3 and n=2 with F4), and 6 HCCs (n=1 with F0, n=2 with F2–3, and n=3 with F4) (Table 1). Across the samples, we analyzed 1,311 FOVs with 3,318 genes/FOV and identified 1,159,260 cells (Supplemental Table S4, https://links.lww.com/HC9/C448). CNV of 5 out of 6 samples demonstrated expected amplifications and deletions in selected known HCC marker genes, namely *MET*, *MYC*, and *CCND1*, among others (Figure 2A),^27^ confirming our spatial analysis samples are HCC, which is corroborated by the histopathologies of the tumors of the resected livers. Regardless of cirrhosis status, HCC cells were smaller, consistent with increased cellular proliferation (184.0 µm^2^ vs. 264.4 µm^2^, *p*=0.0087), but there was no significant change in cell number (126,802 vs. 103,290 cells) (Supplemental Figure S5, https://links.lww.com/HC9/C448, and Supplemental Table S3, https://links.lww.com/HC9/C448).

To determine if zonation is maintained in bioarchived human liver samples, a normal liver (BS217, Table 1) was used as a control to validate zonation marker genes (Supplemental Figure S6, https://links.lww.com/HC9/C448).^28^^,^^29^ Based on our snRNAseq data and availability of marker genes in the CosMx panel, we used *HAMP* (hepcidin antimicrobial peptide) for zone 1–2 hepatocytes and *GLUL* (glutamine synthetase) for zone 3. We confirmed that *GLUL* was predominantly expressed around the central vein and *HAMP* was expressed in zones 1–2 (Supplemental Figures S6C, D, https://links.lww.com/HC9/C448), which was consistent with protein levels of GLUL and HAMP by IHC (Supplemental Figures S6E, F, https://links.lww.com/HC9/C448). In agreement with our snRNAseq analyses, spatial imaging demonstrated that hepatocytes emerged as the predominant cell type (apart from BS460 adjacent non-tumor) (Figure 2B). As expected, pre-cirrhotic adjacent tissue samples showed *GLUL* expression in zone 3 and *HAMP* expression in zones 1–2 by both spatial imaging and IHC validation of adjacent non-tumor control regions. However, the HCC samples predominantly lacked clear GLUL localization by CosMx and IHC, consistent with loss of zonation in HCC (Supplemental Figures S7, S8, https://links.lww.com/HC9/C448). Of note, HSCs were predominantly peritumoral in HCCs of two pre-cirrhotic samples (BS414 and BS460), which directly overlapped with fibrous tissue as confirmed by H&E (Supplemental Figures S7, S8, https://links.lww.com/HC9/C448). In addition, 2 cirrhotic samples, BS630 and BS644, also had peritumoral pseudocapsules seen by H&E and high HSCs subpopulations observed via spatial imaging (Supplemental Figures S10, S11, https://links.lww.com/HC9/C448). Zonation of the cirrhotic tissues for both adjacent non-tumor and HCC samples was also disrupted, particularly in zone 3, with the liver architecture mainly consisting of fibrotic tissue (Supplemental Figures S10–S13, https://links.lww.com/HC9/C448).

To characterize HCC-HSCs spatially further as a function of the cirrhosis status, we selected a pre-cirrhotic (BS414, F2) (Figure 2C) and a cirrhotic (BS644, F4) (Figure 2I) HCC sample. The corresponding H&Es revealed that these samples both contained a peritumoral pseudocapsule that was not present in the adjacent tissue (Figures 2C, I; and Supplemental Figures S7A, S11A, https://links.lww.com/HC9/C448). Overlaying spatial imaging over H&Es confirmed that these pseudocapsules contained a high population of HSCs regardless of cirrhosis status (Figures 2C, D, I, J). Based on this observation and our snRNAseq findings that identified YAP-effector gene upregulation in HSC-HCCs, we investigated the localization of *CCN1* and *CCN2* in our spatial imaging data. We classified the 2 HCC tissues into 3 sections (Figures 2E, K): (A) adjacent non-tumor (not present in BS644-HCC), (B) peritumoral pseudocapsule, and (C) HCC. We calculated HSC subpopulations between the different sections and found that the peritumoral pseudocapsule for both samples contained the highest number of HSCs (Supplemental Figures S7N, S11N, https://links.lww.com/HC9/C448). Consistently, gene expression of *CCN1* was significantly increased by 1.3-fold (*p*=0.0055), and *CCN2* was also significantly increased by 1.3-fold (*p*=0.0004) only in the pseudocapsule of the pre-cirrhotic sample (BS414), relative to the adjacent non-tumor control after normalization to the HSCs cell count per FOV (Figures 2E–H, K–N).

To spatially resolve the subpopulation of HSCs identified by snRNAseq, we assessed the expression of validated marker genes available in CosMx for aHSCs (*IGFBP7*, *ACTA2*, *TIMP1*) and qHSCs (*NTRK2*, *CCL2*, and *HHIP*). This revealed that activated myofibroblast-like HSCs were the predominant subpopulation expressed in the pseudocapsule (Supplemental Figure S14, https://links.lww.com/HC9/C448), consistent with the fibrous band seen on H&E (Figures 2C, I). While present, *NTRK2*, *CCL2*, and *HHIP* were not the predominant genes in the pseudocapsule (Supplemental Figure S14, https://links.lww.com/HC9/C448). The increase in aHSCs marker genes (*IGFBP7*, *ACTA2*, *TIMP1*) and the functional increase in YAP signaling (*CCN1* and *CCN2* effector genes) colocalized to the pseudocapsule suggests this population of aHSCs likely influences the HCC microenvironment.

### Statins downregulate YAP signaling in activated human primary HSCs

Given that statins are being investigated prospectively for HCC chemoprevention, the observation that YAP-effector genes are upregulated only in HCC-HSCs, and previous work demonstrating statins’ ability to regulate YAP, we assessed the associations between the statin-responsive cholesterol pathway genes and YAP activation in human livers.

**FIGURE 3:**
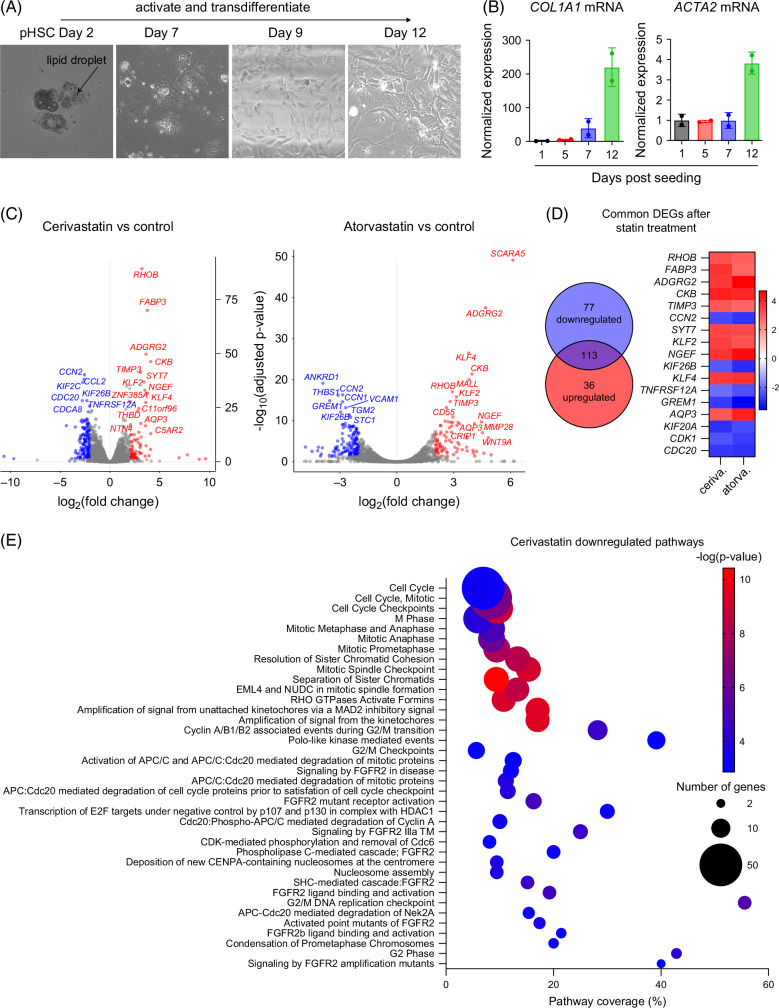
Human bulk RNA sequencing of activated pHSCs confirms downregulation of YAP and Rho with statins. (A) Brightfield images of pHSCs from donor 1 show activation with time post-seeding. (B) pHSCs increase their expression of α-SMA (*ACTA2*) and collagen type 1 (*COL1A1*) from days 1 to 12, relative to day 1; n=2 technical replicates. (C) Volcano plot representations of significantly upregulated and downregulated genes of pHSCs from the 4 donors treated with 1 µM cerivastatin or 2 donors treated with 20 µM atorvastatin for 24 hours in 0.5% FBS, compared with vehicle control. (D) Venn diagram representation of significant DEGs (log_2_FoldChange <−2, *FDR*<0.05). Heatmap of the top 15% of the 113 common DEGs shows similarity between the cerivastatin and atorvastatin treatment. (E) Downregulated pathways with cerivastatin treatment by Reactome analysis reveal the importance of Rho as well as cytoskeleton regulation. Abbreviations: ACTA2, actin alpha 2, smooth muscle; *COL1A1*, collagen type I alpha; DEGs, differentially expressed genes; FBS, fetal bovine serum; FDR, false discovery rate; pHSCs, primary human hepatic stellate cells; YAP, Yes-associated protein; α-SMA, α-smooth muscle actin.

To recapitulate a cirrhosis phenotype, in the background of which most MASLD-HCCs occur, we sought to conduct our experiments in activated human primary HSCs. Our in vitro culture model confirmed that human primary qHSCs lose their lipid droplets and transdifferentiate into myofibroblast-like cells by day 12 post-seeding (Figure 3A and Supplemental Figure S15, https://links.lww.com/HC9/C448). Gene expression analyses confirmed an increase in α-SMA (*ACTA2*) and collagen type I alpha I chain (*COL1A1*) on day 12 compared with days 1–7 (Figure 3B), consistent with an activated state. To identify which genes/pathways are statin-regulated in HSCs in an unbiased fashion, we treated activated human primary HSCs from 4 donors with statins and compared gene expression to vehicle controls (Supplemental Table S5, https://links.lww.com/HC9/C448). Using bulk RNAseq of primary HSCs treated with cerivastatin (n=4) and atorvastatin (n=2) for 24 hours after 11 days of culture, we identified 113 significant DEGs that were common to both statins, with 77 common genes downregulated with statin treatment and 36 common genes that were upregulated (Figures 3C, D, Supplemental Figure S16, https://links.lww.com/HC9/C448, and Supplemental Tables S6, S7, https://links.lww.com/HC9/C448). *CCN1* and *CCN2* had significantly decreased expression in all 4 donors with both statins compared with control aHSCs, consistent with (1) our snRNAseq and spatial imaging findings that YAP-target gene expression is upregulated in HSC, and (2) that statins downregulate YAP activity (Figures 3C, D and Supplemental Tables S6, S7, https://links.lww.com/HC9/C448). In addition, we found that statins significantly downregulated Rho GTPases, cell cycle, and mitosis gene pathways, pathways that were upregulated in our HCC snRNAseq dataset (Figure 3E and Supplemental Figure S16, https://links.lww.com/HC9/C448). These findings, in tandem with our snRNAseq data, led us to further explore the relationship between Rho GTPases and YAP activity in the regulation of HSCs by statins.

### YAP nuclear localization in HSCs is regulated by statins

**FIGURE 4:**
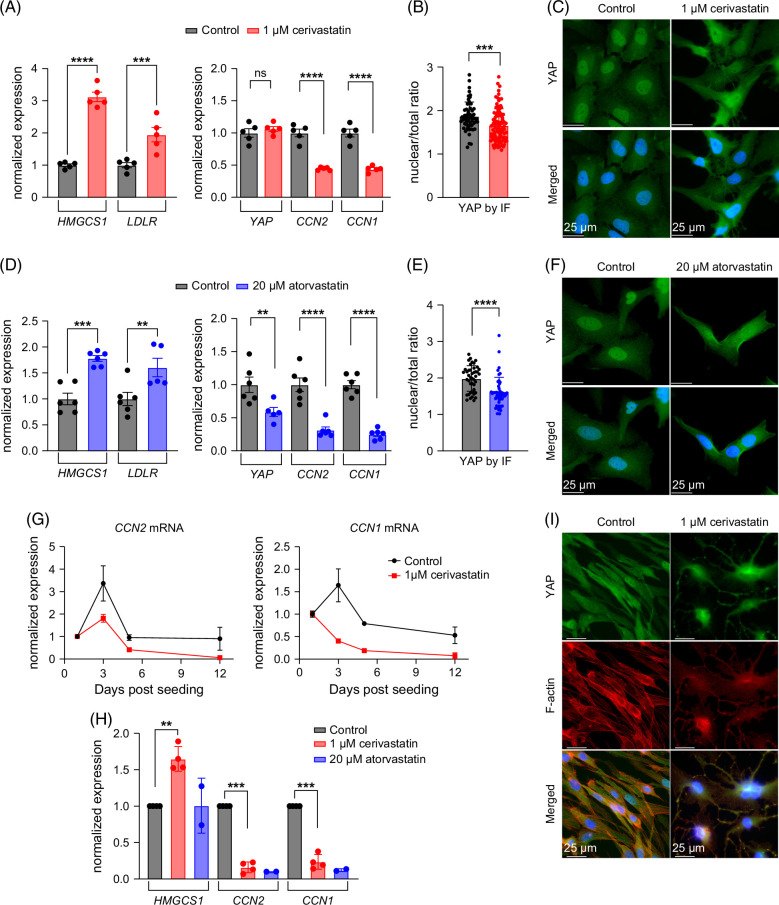
Lipophilic statins regulate YAP protein localization in HSCs in vitro. (A) LX2 cells were treated for 24 hours with 1 µM cerivastatin in 0.5% FBS. *HMGCS1* and *LDLR* mRNA levels increased while downstream effector genes *CCN1* and *CCN2* decreased by qPCR, compared with an untreated control; n=5, ***p=0.0004, and ****p<0.0001 by one-way ANOVA. (B, C) Cerivastatin also led to cytosolic YAP protein retention by IF; n=57–79 and ***p=0.00022 by Mann–Whitney *U* test. (D) LX2 cells were also atorvastatin-responsive with a significant increase in *HMGCS1* and *LDLR* mRNA and a decrease in *CCN1* and *CCN2* expression with 20 µM atorvastatin in 0.5% FBS. n=6, **p<0.0049, ***p=0.0003, and ****p<0.0001 by one-way ANOVA. (E, F) A 24-hour treatment with 20 µM atorvastatin in 0.5% FBS also led to cytosolic YAP protein retention by IF (n=43–49 and ****p<0.0001 by Mann–Whit*n*ey *U* test). (G) Primary human HSCs from donor 1 were treated with cerivastatin before, during, and after activation from days 1 to 12 post-seeding and compared with vehicle control. *CCN1* and *CCN2* expression levels are highest on day 3, with the primary HSCs’ response to cerivastatin dampened with each time post-seeding. (H) Primary HSCs were plated and grown for 12 days before cerivastatin (n=4; donors 1–4) treatment and atorvastatin (n=2; donors 3–4) for 24 hours in 0.5% FBS. *HMGSC1* mRNA is significantly increased with both statins, confirming that primary HSCs are statin-responsive. *CCN1* and *CCN2* expressions were reduced for both treatments, implicating YAP in pHSCs. *p* values were calculated for 1 µM cerivastatin treated pHSCs only; n=4, **p=0.0046, and ***p<0.0006 by the Student *t* test. (I) Primary HSCs from donor 1 were grown to day 12 before 24 hours of cerivastatin treatment, which led to the cytoskeleton structure of primary HSCs. Abbreviations: ANOVA, analysis of variance; CCN1, cellular communication factor 1; CCN2, cellular communication factor 2; FBS, fetal bovine serum; HSCs, hepatic stellate cells; IF, immunofluorescence; LDLR, low-density lipoprotein receptor; mRNA, messenger RNA; pHSCs, primary human hepatic stellate cells; qPCR, quantitative polymerase chain reaction; YAP, Yes-associated protein; α-SMA, α-smooth muscle actin.

To begin to test the involvement of YAP in HSC activation and how this regulatory axis is impacted by statins, we examined YAP function in human LX2 and primary HSCs. Cells were plated at low cellular density to prevent Hippo pathway activation, which leads to YAP nuclear extrusion, since we resolved that YAP is regulated in HSCs (Supplemental Figure S17, https://links.lww.com/HC9/C448). LX2 cells treated with 1 µM cerivastatin led to the expected significant increase in *HMGCS1* and *LDLR* mRNA levels (Figure 4A), which was mirrored at 20 µM atorvastatin (Figure 4D), consistent with statin-induced positive feedback loop leading to increased SREBP2-dependent cholesterol pathway genes.^30^ We saw through dose–response gene expression that the atorvastatin effects began at 7.5 µM, with a higher dose needed to recapitulate the cerivastatin response (Supplemental Figures S18A, B, https://links.lww.com/HC9/C448), without cytotoxicity (Supplemental Figure S18C, https://links.lww.com/HC9/C448). Brightfield images of LX2 cells after atorvastatin treatment revealed stark cellular cytoskeleton changes at 20 µM (Supplemental Figure S18D, https://links.lww.com/HC9/C448), suggesting a statin-mediated cytoskeletal restructuring, which is known to impact YAP function. Consequently, we observed decreased YAP protein nuclear localization by IF and decreased expression of its effector genes *CCN1* and *CCN2* with cerivastatin and atorvastatin (Figures 4A–F). Since transcriptional co-Activator with PDZ-binding motif (TAZ), a homolog of YAP, is also upstream of *CCN1* and *CCN2*, we visualized and quantified TAZ protein localization by IF to understand its regulation after statin treatment. Although we saw a slight decrease in nuclear localization of TAZ after cerivastatin treatment, the effect was not significant (Supplemental Figure S18E, https://links.lww.com/HC9/C448) and was not affected by exogenous GGPP. Taken together, these data indicate that HSCs are statin-responsive and that statins block YAP nuclear localization in HSCs, thereby downregulating downstream YAP-effector gene expression in these cells.

To determine the time-course of YAP regulation by statins in primary HSCs, cells were treated with cerivastatin, and the gene expression of *CCN1* and *CCN2* was assessed before, during, and after activation. The expression of YAP-TEAD-regulated genes peaked on day 3 post-seeding, when YAP is nuclear; however, statin exposure markedly blunted the induction of YAP-TEAD-regulated genes on day 3, and this effect was maintained until day 12 (Figure 4G). To mirror current clinical trials of statin chemoprevention where patients have cirrhosis and fully activated HSCs, we chose to investigate the statin effect in primary HSCs at day 12. Primary human HSCs were treated with cerivastatin (donors 1–4) or atorvastatin (donors 3–4) on day 11 before isolating RNA on day 12. Primary HSCs are statin responsive, as seen by the upregulation of *HMGCS1* for all donors treated with either statin and downregulation of both *CCN1* and *CCN2* expression, replicating the statin effects seen in cultured LX2 cells (Figure 4H). Furthermore, treatment of primary HSCs induced a striking cytoskeletal restructuring (Figure 4I), confirming that statins regulate YAP localization in both LX2 cells and human primary HSCs, likely through cytoskeleton reorganization.

### GGPP leads to YAP nuclear localization

**FIGURE 5:**
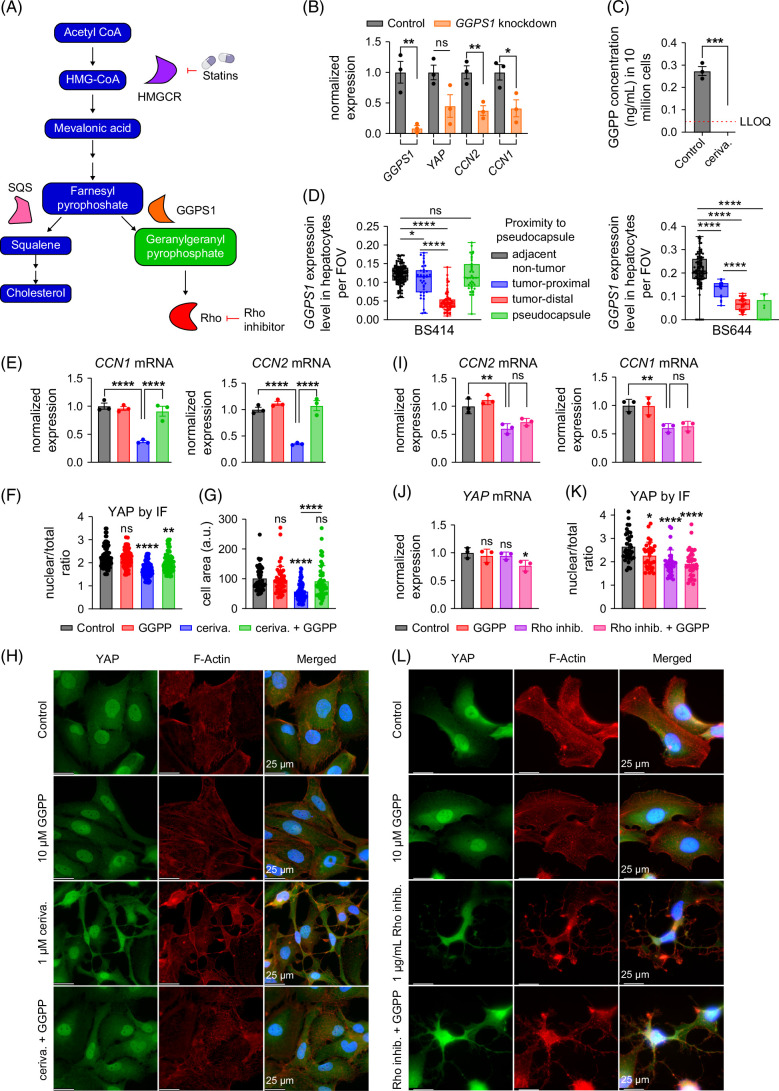
Exogenous GGPP replacement reverses statin effects on YAP. (A) Schematic representation of a simplified mevalonate pathway showing key metabolites and enzymes targeted for this study. (B) Knockdown of *GGPS1* (GGPP synthase) with siRNA for 48 hours in 0.5% FBS led to ~90% decreased in *GGPS1* mRNA levels. This caused a decreased expression of *CCN1* and *CCN2* mRNA levels; **p<0.0092 and *p<0.0347 by the Student *t* test. (C) Cerivastatin reduced the intracellular level of GGPP in LX2s below the lower limit of quantification (LLOQ) by UPLC-MS/MS significantly; n=3 and ***p=0.0004 by the Student *t* test. (D) Further segmentation of BS414-HCC and BS644-HCC relative to the proximity of the pseudocapsule revealed that tumor hepatocytes had a higher *GGPS1* expression proximal to the pseudocapsule compared with the distal tumor hepatocytes. FOVs n=9–124, *p=0.0420, and ****p<0.0001. (E) LX2 cells were treated with either GGPP, cerivastatin, or a combination for 24 hours in 0.5% FBS. Cerivastatin altered CCN1 and CCN2 mRNA levels, and while GGPP alone does not affect the expression of the YAP-regulated genes, it recovered CCN1 and CCN2 to baseline levels when combined with cerivastatin (n=3). Not labeled, but there was no significant change between vehicle control and 10 µM GGPP, as well as vehicle control and 1 µM cerivastatin +GGPP, on the expression of *CCN1* and *CCN2*; ****p<0.0001 by one-way ANOVA. (F–H) YAP protein localization and cytoskeleton changes were visualized by IF. YAP protein was retained in the cytosol, and the cellular area shrank after 24 hours of treatment with cerivastatin in 0.5% FBS, which could be recovered with GGPP treatment; n=48–67, **p=0.0015, and ****p<0.0001 by one-way ANOVA. Identifiers of significance are all relative to control. (I, J) LX2 cells were treated for 4 hours with an irreversible Rho inhibitor, and the Rho inhibitor did not alter *YAP* expression but significantly decreased the YAP-TEAD genes. GGPP supplementation did not recover the statin-mediated decrease in gene expression, n=3. Not labeled, but there was no significant change between the vehicle control and 10 µM GGPP; *p<0.0442 and **p<0.0065, by one-way ANOVA. (K, L) YAP protein localization and cytoskeleton changes were visualized, and the YAP nuclear/total ratio was quantified with Rho inhibitor treatment. GGPP supplementation did not reverse the YAP cytoplasmic retention or the cytoskeleton shrinkage; n=31–45, *p=0.0142, and ****p<0.0001 by one-way ANOVA. Identifiers of significance are all relative to control. Not shown on the figure, but there was no significant difference between Rho inhibitor treatment and Rho inhibitor +10 µM GGPP by the Mann–Whitney *U* test. Abbreviations: ANOVA, analysis of variance; CCN1, cellular communication factor 1; CCN2, cellular communication factor 2; FBS, fetal bovine serum; FOVs, fields of view; GGPS1, GGPP synthase 1; GGPP, geranylgeranyl pyrophosphate; HCC, hepatocellular carcinoma; IF, immunofluorescence; LLOQ, lower limit of quantification; siRNA, small interfering RNA; UPLC-MS/MS, ultra-high-performance liquid chromatography–tandem mass spectrometry; YAP, Yes-associated protein.

To determine how statins downregulate YAP activity in HSCs, we examined whether this effect is mediated by the suppression of mevalonate pathway metabolites. Statins inhibit 3-hydroxy-3-methylglutaryl-CoA reductase (HMGCR), the rate-limiting step in the biosynthesis of mevalonic acid. Mevalonic acid subsequently generates farnesyl pyrophosphate (FPP) (Figure 5A), which in turn gives rise to squalene via squalene synthase (SQS), the first dedicated enzyme of the cholesterol pathway,^31^ and GGPP, synthesized by geranylgeranyl diphosphate synthase 1 (GGPS1). Geranylgeranyltransferase type I (GGTase-I) utilizes the lipid moiety of GGPP to prenylate membrane-associating proteins, including Rho (Figure 5A), allowing them to become membrane-bound and active for signal transduction.^32^ Consequently, increases in GGPP will expectably activate Rho. Since we observed the upregulation of Rho GTPases in HCC-HSCs in our human snRNAseq dataset (Figure 1E), and the known regulation of YAP by Rho,^33^ and considering our data showing the inhibition of YAP function by atorvastatin is dose-dependent (Supplemental Figures S18A, B, https://links.lww.com/HC9/C448), we hypothesized that statins downregulate YAP activity by reducing levels of a lipid species generated via the mevalonate pathway. Specifically, since Rho prenylation leads to cytoskeletal changes and YAP nuclear translocation/activity, we hypothesized that decreases in GGPP levels reduce Rho prenylation, lead to cytoskeletal changes and YAP nuclear activity, thereby explaining the chemoprotective benefit of statins for HCC.

To test this possibility, we first performed a siRNA knockdown of *GGPS1* to reduce GGPP synthesis and determine the effect of lowering GGPP levels on YAP nuclear localization and YAP-target gene expression. With ~90% knockdown of *GGPS1*, we observed markedly decreased expression of both YAP targets *CCN1* and *CCN2* (Figure 5B), indicating that the GGPS1 product, GGPP, likely drives YAP nuclear localization and expression of its effector genes in HSCs. Consistently, upon measuring intracellular levels of GGPP in LX2 cells, we observed a depletion of GGPP (undetectable levels; <0.05 ng/mL, n=3) in response to cerivastatin and atorvastatin treatment when compared with vehicle-treated controls (0.27±0.02 ng/mL, 0.14±0.03 ng/mL, n=3) (Figure 5C and Supplemental Figure S19A, https://links.lww.com/HC9/C448).

### Hepatocytes contribute to GGPP levels in the activation of HSC YAP signaling

Given these findings, we revisited our human spatial imaging data of HCC BS414 (pre-cirrhotic) and BS644 (cirrhotic) and found that *GGPS1* was primarily expressed in hepatocytes [average *GGPS1* transcript count in BS644-HCC: n=52.04 in hepatocytes vs. 23.05 in all other cell types (*p*<0.0001) and BS414-HCC: n=64.85 in hepatocytes vs. 18.52 in all other cells (*p*<0.0001)]. When we spatially resolved its expression pattern, we found that *GGPS1* expression in tumor hepatocytes was markedly reduced distal to the pseudocapsule, with relatively higher expression proximal to the pseudocapsule, where most subpopulations of HSCs are localized (Figure 5D and Supplemental Figure S20, https://links.lww.com/HC9/C448). We hypothesize that the spatial distribution of *GGPS1,* which is enriched in peritumoral HCCs, leads to increased synthesis of GGPP, which may activate HSCs in the pseudocapsule region. This possibly reflects a previously undescribed hepatocyte-HSC crosstalk wherein increased GGPP synthesis in tumor hepatocytes may influence HSC activation.

Due to the elevated expression of *GGPS1* in human tumor hepatocytes proximal to the peritumoral pseudocapsule compared with distal tumor hepatocytes, we hypothesized that exogenous GGPP repletion after statin treatment in HSCs would reverse the YAP effects. As expected, GGPP supplementation completely reversed the inhibitory effects of statins on YAP, but not TAZ, nuclear localization, and YAP-target gene expression in LX2 cells (Figure 5E, Supplemental Figures S18E, https://links.lww.com/HC9/C448, S19B, C, https://links.lww.com/HC9/C448). Finally, statin-induced cytosolic retention of YAP protein, as well as changes in cytoskeletal organization assessed by F-actin, were partially reversed by exogenous administration of GGPP (Figures 5F–H and Supplemental Figures S19D, E, https://links.lww.com/HC9/C448). Taken together, these findings show that statins lead to YAP protein cytosolic retention and dampen HSC activation through reduction of intracellular GGPP levels.

To test that GGPP is a key modulator of YAP localization via Rho prenylation, we tested the effect of a GGTase-I inhibitor and a direct Rho inhibitor on YAP nuclear localization and expression of YAP-target genes. LX2 cells were treated with BAY-593, a lead compound shown to inhibit YAP by targeting GGTase-I.^34^ Inhibiting GGTase-I prevented GGPP prenylation, causing: (1) decreased expression of *CCN1* and *CCN2*, (2) the retention of YAP in the cytosol, and (3) cytoskeleton restructuring (Supplemental Figures S21A–E, https://links.lww.com/HC9/C448). We also confirmed the results were not due to general cytotoxicity and cell death (Supplemental Figure S21F, https://links.lww.com/HC9/C448). The Rho inhibitor treatment, a cell-permeable C3 transferase that inactivates Rho but does not affect related GTPases, mimicked the YAP effects seen with statins and significantly decreased the expression of *CCN1* and *CCN2* without altering *YAP* expression and led to cytosolic YAP retention and stark cytoskeleton changes, similarly to those seen in statin-treated LX2 cells (Figures 5I–L). Expectedly, these changes were not reversed with GGPP (Figures 5I–L).

## DISCUSSION

A comprehensive characterization of the intrahepatic cell types is essential for understanding the pathogenesis of MASLD-HCC and its microenvironment. This study presents one of the largest transcriptomic studies of MASLD-HCC livers to date, leveraging matched snRNAseq and spatial imaging analyses, identifying 265,403 nuclei and 1,159,260 single cells, respectively. We: (1) resolve HSC subclusters in HCCs of cirrhotic, pre-cirrhotic, and non-cirrhotic livers and identify an HSC-HCC specific aHSC+dHSC phenotype; (2) demonstrate that HCC-related HSCs are predominantly localized in the peritumoral pseudocapsule, independently of cirrhosis status, and significantly upregulate the YAP pathway; (3) show that tumor hepatocytes likely contribute to GGPP synthesis, which may activate proximal HSCs in the pseudocapsule; and (4) show that statins decrease intracellular GGPP levels, leading to decreased Rho GTPases and YAP activity.

Pathway analysis of DEGs uncovered by snRNAseq revealed an inverse relationship with Rho GTPases in HSCs and hepatocytes of HCCs, suggesting cellular communication between HSCs, the key drivers of hepatic fibrosis, and hepatocytes, the HCC cell type of origin.^35^ Our spatial imaging data support that GGPP is primarily synthesized in hepatocytes (given the increased *GGPS1* expression in hepatocytes), which may lead to HSC activation. In addition, HSCs, along with LSECs, in the HCCs were the only 2 cell types to have an upregulation of YAP-TEAD genes, pointing to a key relationship between HCC, Rho GTPases, and YAP in the tumor microenvironment. Previous work from our group and others has identified YAP as a key pathway in HCC pathogenesis;^23^^,^^24^ however, the cell-type-specific effects in the spectrum of disease remained unclear. Our new findings in this study further shed light on the function of YAP in HSCs and its role in the larger progression of MASLD to HCC.

Leveraging spatial molecular imaging allowed for the spatial characterization of intact, FFPE tissues, giving us a sweeping view of MASLD-HCC liver architecture. We focused predominantly on 1 pre-cirrhotic and 1 cirrhotic sample to further investigate HSCs in the HCC tumor microenvironment and the role of the YAP pathway. Our work revealed an intricate environment between the tumor, peritumoral pseudocapsule, and adjacent non-tumor cells. We found that YAP signaling was increased in HSCs localized in the pseudocapsule in pre-cirrhosis but not in cirrhosis HCC. Interestingly, while HSCs were in the pseudocapsule of both cirrhosis and pre-cirrhosis livers, they were mainly localized to the region closest to the non-tumor tissue. Consistently, we also saw that tumor-specific *GGPS1* expression was the highest around the pseudocapsule, where HSCs localize, suggesting that HSC activation and pseudocapsule formation may be independent of underlying cirrhosis. Consistent with Filiol et al’s mouse models, we also found that most HSCs were less abundant in the HCCs, compared with adjacent non-tumor controls. However, we identified an HSC subpopulation enriched in HCCs, consisting of a heterogeneous and mixed population of aHSCs and dHSCs, involved in ECM remodeling.^20^^,^^21^ Other HSC subtypes, including qHSCs, were downregulated in subcluster 1, the predominant subcluster in HCCs, while inflammatory HSCs and senescent HSCs were not distinctly identified.^21^^,^^22^ Taken together, these results indicate that HCC-HSCs are dynamic and may remodel the tumor microenvironment, perhaps to evade immune surveillance.

Comparing the three liver sections (tumor, pseudocapsule, and adjacent non-tumor) spatially, we observed that aHSCs in the pseudocapsule colocalized with inflammatory macrophages, NK cells, and plasma cells, which can be attributed to the preservation of the immune environment that is normally lost with tissue dissociation in snRNAseq.^28^ Since chronic liver disease leads to HSC activation, ECM deposition (through YAP activity), and eventually cirrhosis, the structural changes that result from these effects reshape the checks and balances set by the immune system. These play a role in tumor progression through several previously reported mechanisms, including by *CCN1*-mediated suppression of T-cell proliferation,^36^^,^^37^ decreasing of tumor derived antigens that normally reside on the ECM,^38^^,^^39^ and impaired NK cell activity.^40^ While understanding the HSC-immune crosstalk is beyond the scope of these studies, our aHSC co-localization with other immune cells suggests an intricate and important cellular communication between immune cells and HSCs, which necessitates further investigations.

Lipophilic statins have been shown to have potential anti-cancer properties,^41^ including in HCC, where statin use is associated with a decreased HCC incidence and improved overall survival.^9^^,^^42^^,^^43^ These mechanisms are beginning to be investigated, as our work and others have shown that statins inhibit YAP activity in HCC and several other tumor types.^5^^,^^24^^,^^44^ The HSC-HCC upregulation of YAP-effectors genes in our human snRNAseq data suggests that YAP and its downstream effectors are critical to HCC and its microenvironment, beyond their role in cirrhosis. Since statins also have an immune modulatory role,^45^^,^^46^ and given that HSCs colocalized to NK cells and other immune cells in the human peritumoral HCCs by spatial imaging, we hypothesize that the statin-mediated YAP inhibition may lead to decreased HSC activity, potentially leading to increased tumor immune cell infiltration and response to therapy. Bulk RNAseq of human primary aHSC further confirms our human snRNAseq and spatial imaging datasets and solidifies that statins target Rho GTPases and decrease YAP activity, contributing to their chemoprotective effects. While we could not determine how statin exposure affected other cell types in our human transcriptomic analyses, future studies are needed to determine if HSCs or other cell types are the main cell type targeted by statins. Our detailed stepwise, mechanistic work also demonstrates that the statin-mediated YAP effects in the human primary HSCs are dampened once active (since YAP is at its greatest activity before HSC activation).^25^ These data suggest that statins would affect different biological pathways^22^ in the inactivated HSCs compared with their fully active states.

Taken together, our large, detailed human snRNAseq with matched spatial imaging analyses, compounded with our human primary aHSCs and LX2 in vitro experiments, show that YAP activity is key in HCC-HSCs and likely shapes the HCC microenvironment, which may be modified by statins through GGPP. This provides critical insight into the function and regulation of HSCs since they are the key cell type of cirrhosis onset to HCC progression. We characterized HSC-specific YAP activity and how statins, and specifically GGPP, regulate YAP function, likely through Rho GTPases cell signaling. Our findings support the need to further study how statins and GGPP can potentially be used to target ECM remodeling in HCCs and enhance immune surveillance.

## Supplementary Material

## References

[1] Younossi ZM Paik JM Henry L Yang J Fernandes G Stepanova M. The growing economic and clinical burden of nonalcoholic steatohepatitis (NASH) in the United States. J Clin Exp Hepatol. 2023;13:454–467. 37250870 10.1016/j.jceh.2022.12.005 PMC10213853

[2] Younossi Z Stepanova M Ong JP Jacobson IM Bugianesi E Duseja A. Nonalcoholic steatohepatitis is the fastest growing cause of hepatocellular carcinoma in liver transplant candidates. Clin Gastroenterol Hepatol. 2019;17:748–755.e3. 29908364 10.1016/j.cgh.2018.05.057

[3] Qiu S Cai J Yang Z He X Xing Z Zu J. Trends in hepatocellular carcinoma mortality rates in the US and projections through 2040. JAMA Netw Open. 2024;7:e2445525. 39556395 10.1001/jamanetworkopen.2024.45525 PMC11574689

[4] Sung H Ferlay J Siegel RL Laversanne M Soerjomataram I Jemal A. Global Cancer Statistics 2020: GLOBOCAN estimates of incidence and mortality worldwide for 36 cancers in 185 countries. CA Cancer J Clin. 2021;71:209–249. 33538338 10.3322/caac.21660

[5] Filliol A Saito Y Nair A Dapito DH Yu LX Ravichandra A. Opposing roles of hepatic stellate cell subpopulations in hepatocarcinogenesis. Nature. 2022;610:356–365. 36198802 10.1038/s41586-022-05289-6 PMC9949942

[6] Benhammou JN Lin J Aby ES Markovic D Raman SS Lu DS. Nonalcoholic fatty liver disease-related hepatocellular carcinoma growth rates and their clinical outcomes. Hepatoma Res. 2021;7:70. 34966854 10.20517/2394-5079.2021.74 PMC8713558

[7] Kanwal F Kramer JR Mapakshi S Natarajan Y Chayanupatkul M Richardson PA. Risk of hepatocellular cancer in patients with non-alcoholic fatty liver disease. Gastroenterology. 2018;155:1828–1837.e2. 30144434 10.1053/j.gastro.2018.08.024 PMC6279617

[8] Singal AG Llovet JM Yarchoan M Mehta N Heimbach JK Dawson LA. AASLD Practice Guidance on prevention, diagnosis, and treatment of hepatocellular carcinoma. Hepatology. 2023;78:1922–1965. 37199193 10.1097/HEP.0000000000000466 PMC10663390

[9] Kaplan DE Serper MA Mehta R Fox R John B Aytaman A. Effects of hypercholesterolemia and statin exposure on survival in a large national cohort of patients with cirrhosis. Gastroenterology. 2019;156:1693–1706.e12. 30660733 10.1053/j.gastro.2019.01.026

[10] Lee YA Wallace MC Friedman SL. Pathobiology of liver fibrosis: A translational success story. Gut. 2015;64:830–841. 25681399 10.1136/gutjnl-2014-306842 PMC4477794

[11] Tsuchida T Friedman SL. Mechanisms of hepatic stellate cell activation. Nat Rev Gastroenterol Hepatol. 2017;14:397–411. 28487545 10.1038/nrgastro.2017.38

[12] SchwabeRFBrennerDA. Hepatic stellate cells: Balancing homeostasis, hepatoprotection and fibrogenesis in health and disease. Nat Rev Gastroenterol Hepatol. 2025;22:481–499.40404839 10.1038/s41575-025-01068-6

[13] Kamm DR McCommis KS. Hepatic stellate cells in physiology and pathology. J Physiol. 2022;600:1825–1837. 35307840 10.1113/JP281061 PMC9012702

[14] Kato M Iwamoto H Higashi N Sugimoto R Uchimura K Tada S. Role of Rho small GTP binding protein in the regulation of actin cytoskeleton in hepatic stellate cells. J Hepatol. 1999;31:91–99. 10424288 10.1016/s0168-8278(99)80168-8

[15] Ji J Eggert T Budhu A Forgues M Takai A Dang H. Hepatic stellate cell and monocyte interaction contributes to poor prognosis in hepatocellular carcinoma. Hepatology. 2015;62:481–495. 25833323 10.1002/hep.27822 PMC4515211

[16] Wang S Li K Pickholz E Dobie R Matchett KP Henderson NC. An autocrine signaling circuit in hepatic stellate cells underlies advanced fibrosis in nonalcoholic steatohepatitis. Sci Transl Med. 2023;15:eadd3949. 36599008 10.1126/scitranslmed.add3949 PMC10686705

[17] Sugimoto A Saito Y Wang G Sun Q Yin C Lee KH. Hepatic stellate cells control liver zonation, size and functions via R-spondin 3. Nature. 2025;640:752–761. 40074890 10.1038/s41586-025-08677-w PMC12003176

[18] Brunt EM Kleiner DE Wilson LA Belt P Neuschwander-Tetri BA. Nonalcoholic fatty liver disease (NAFLD) activity score and the histopathologic diagnosis in NAFLD: Distinct clinicopathologic meanings. Hepatology. 2011;53:810–820. 21319198 10.1002/hep.24127 PMC3079483

[19] Alvarez M Benhammou JN Darci-Maher N French SW Han SB Sinsheimer JS. Human liver single nucleus and single cell RNA sequencing identify a hepatocellular carcinoma-associated cell-type affecting survival. Genome Med. 2022;14:50. 35581624 10.1186/s13073-022-01055-5 PMC9115949

[20] Cogliati B Yashaswini CN Wang S Sia D Friedman SL. Friend or foe? The elusive role of hepatic stellate cells in liver cancer. Nat Rev Gastroenterol Hepatol. 2023;20:647–661. 37550577 10.1038/s41575-023-00821-z PMC10671228

[21] Kim HY Rosenthal SB Liu X Miciano C Hou X Miller M. Multi-modal analysis of human hepatic stellate cells identifies novel therapeutic targets for metabolic dysfunction-associated steatotic liver disease. J Hepatol. 2025;82:882–897. 39522884 10.1016/j.jhep.2024.10.044 PMC12306656

[22] Yashaswini CN Qin T Bhattacharya D Amor C Lowe S Lujambio A. Phenotypes and ontogeny of senescent hepatic stellate cells in metabolic dysfunction-associated steatohepatitis. J Hepatol. 2024;81:207–217. 38508241 10.1016/j.jhep.2024.03.014 PMC11269047

[23] Fan W Adebowale K Váncza L Li Y Rabbi MF Kunimoto K. Matrix viscoelasticity promotes liver cancer progression in the pre-cirrhotic liver. Nature. 2024;626:635–642. 38297127 10.1038/s41586-023-06991-9 PMC10866704

[24] Benhammou JN Qiao B Ko A Sinnett-Smith J Pisegna JR Rozengurt E. Lipophilic statins inhibit YAP coactivator transcriptional activity in HCC cells through Rho-mediated modulation of actin cytoskeleton. Am J Physiol Gastrointest Liver Physiol. 2023;325:G239–G250. 37366601 10.1152/ajpgi.00089.2023 PMC10511177

[25] Mannaerts I Leite SB Verhulst S Claerhout S Eysackers N Thoen LFR. The Hippo pathway effector YAP controls mouse hepatic stellate cell activation. J Hepatol. 2015;63:679–688. 25908270 10.1016/j.jhep.2015.04.011

[26] Wang Y Xu X Maglic D Dill MT Mojumdar K Ng PKS. Comprehensive molecular characterization of the hippo signaling pathway in cancer. Cell Rep. 2018;25:1304–1317.e5. 30380420 10.1016/j.celrep.2018.10.001 PMC6326181

[27] Ally A Balasundaram M Carlsen R Chuah E Clarke A Dhalla N. Comprehensive and integrative genomic characterization of hepatocellular carcinoma. Cell. 2017;169:1327–1341.e23. 28622513 10.1016/j.cell.2017.05.046 PMC5680778

[28] Andrews TS Atif J Liu JC Perciani CT Ma XZ Thoeni C. Single-cell, single-nucleus, and spatial RNA sequencing of the human liver identifies cholangiocyte and mesenchymal heterogeneity. Hepatol Commun. 2022;6:821–840. 34792289 10.1002/hep4.1854 PMC8948611

[29] Paris J Henderson NC. Liver zonation, revisited. Hepatology. 2022;76:1219–1230. 35175659 10.1002/hep.32408 PMC9790419

[30] Brown MS Goldstein JL. Multivalent feedback regulation of HMG CoA reductase, a control mechanism coordinating isoprenoid synthesis and cell growth. J Lipid Res. 1980;21:505–517. 6995544

[31] Sitaula S Burris TP. Cholesterol and other steroids Bradshaw RA Stahl PD. Encyclopedia of Cell Biology. Waltham: Academic Press; 2016:173–179.

[32] Zhao Y Wu T-Y Zhao M-F Li CJ. The balance of protein farnesylation and geranylgeranylation during the progression of nonalcoholic fatty liver disease. J Biol Chem. 2020;295:5152–5162. 32139507 10.1074/jbc.REV119.008897 PMC7152775

[33] Totaro A Panciera T Piccolo S. YAP/TAZ upstream signals and downstream responses. Nat Cell Biol. 2018;20:888–899. 30050119 10.1038/s41556-018-0142-z PMC6186418

[34] Graham K Lienau P Bader B Prechtl S Naujoks J Lesche R. Discovery of YAP1/TAZ pathway inhibitors through phenotypic screening with potent anti-tumor activity via blockade of Rho-GTPase signaling. Cell Chem Biol. 2024;31:1247–1263.e16. 38537632 10.1016/j.chembiol.2024.02.013

[35] Mu X Español-Suñer R Mederacke I Affò S Manco R Sempoux C. Hepatocellular carcinoma originates from hepatocytes and not from the progenitor/biliary compartment. J Clin Invest. 2015;125:3891–3903. 26348897 10.1172/JCI77995 PMC4607132

[36] Bian Z Peng Y You Z Wang Q Miao Q Liu Y. CCN1 expression in hepatocytes contributes to macrophage infiltration in nonalcoholic fatty liver disease in mice. J Lipid Res. 2013;54:44–54. 23071295 10.1194/jlr.M026013 PMC3520538

[37] Zhang H Lian M Zhang J Bian Z Tang R Miao Q. A functional characteristic of cysteine-rich protein 61: Modulation of myeloid-derived suppressor cells in liver inflammation. Hepatology. 2018;67:232–246. 28777871 10.1002/hep.29418

[38] Gong D Shi W Yi S Chen H Groffen J Heisterkamp N. TGFbeta signaling plays a critical role in promoting alternative macrophage activation. BMC Immunol. 2012;13:31. 22703233 10.1186/1471-2172-13-31 PMC3406960

[39] Marcoe JP Lim JR Schaubert KL Fodil-Cornu N Matka M McCubbrey AL. TGF-beta is responsible for NK cell immaturity during ontogeny and increased susceptibility to infection during mouse infancy. Nat Immunol. 2012;13:843–850. 22863752 10.1038/ni.2388 PMC3426626

[40] Liu P Chen L Zhang H. Natural killer cells in liver disease and hepatocellular carcinoma and the NK cell-based immunotherapy. J Immunol Res. 2018;2018:1206737. 30255103 10.1155/2018/1206737 PMC6142725

[41] Jiang W Hu JW He XR Jin WL He XY. Statins: A repurposed drug to fight cancer. J Exp Clin Cancer Res. 2021;40:1–33. 34303383 10.1186/s13046-021-02041-2 PMC8306262

[42] Simon TG Duberg AS Aleman S Hagstrom H Nguyen LH Khalili H. Lipophilic statins and risk for hepatocellular carcinoma and death in patients with chronic viral hepatitis: Results from a nationwide Swedish population. Ann Intern Med. 2019;171:318–327. 31426090 10.7326/M18-2753 PMC8246628

[43] Vell MS Loomba R Krishnan A Wangensteen KJ Trebicka J Creasy KT. Association of statin use with risk of liver disease, hepatocellular carcinoma, and liver-related mortality. JAMA Network Open. 2023;6:e2320222. 37358849 10.1001/jamanetworkopen.2023.20222 PMC10293910

[44] Hao F Xu Q Wang J Yu S Chang HH Sinnett-Smith J. Lipophilic statins inhibit YAP nuclear localization, co-activator activity and colony formation in pancreatic cancer cells and prevent the initial stages of pancreatic ductal adenocarcinoma in KrasG12D mice. PLoS One. 2019;14:e0216603. 31100067 10.1371/journal.pone.0216603 PMC6524808

[45] Pham N Benhammou JN. Statins in chronic liver disease: Review of the literature and future role. Semin Liver Dis. 2024;44:191–208. 38701856 10.1055/a-2319-0694

[46] Bekkering S Arts RJW Novakovic B Kourtzelis I van der Heijden CDCC Li Y. Metabolic induction of trained immunity through the mevalonate pathway. Cell. 2018;172:135–146.e9. 29328908 10.1016/j.cell.2017.11.025

