# Supplementary material for: Hepatic stellate cell subpopulations in human MASLD-related hepatocellular carcinoma upregulate YAP via geranylgeranyl pyrophosphate
Source: Hepatol Commun. 2026 Aug 14;10(9):e1015. doi: 10.1097/HC9.0000000000001015 (PMC13480919; doi:10.1097/HC9.0000000000001015)
Supplement: Supplementary file 1 [file hc9-10-e1015-s001.pdf]

Hepatic stellate cell subpopulations in human MASLD-related hepatocellular carcinoma upregulate YAP via geranylgeranyl pyrophosphate

Courtney L. Labrecque[1,2,3], Arthur Ko[4], Bo Qiao[1], Calvin Pan[2,3], Renu Nandakumar[5], , Kushan Chowdhury[2,6], Namrata Venkatesan[1], Xiaotang Du[7], Bishuang Cai[2,6], Matteo Pellegrini[3,8], Joseph R. Pisegna[1,2,3], Samuel W. French[7,3], Vatche Agopian[3,9,10], Enrique Rozengurt[2,6], Rajat Singh[2,3,6], Jihane N. Benhammou[1,2,3,10]

[1]Veterans Affairs, Greater Los Angeles Healthcare System, Los Angeles, CA

[2]David Geffen School of Medicine, University of California, Los Angeles, Los Angeles, CA

[3]Comprehensive Liver Research Center, University of California, Los Angeles, Los Angeles, CA

[4]Center for Precision Medicine and Genomics Research, Children's National Research Institute, Washington, DC

[5]Columbia University Biomarkers Core Laboratory, New York, NY

[6]Vatche and Tamar Manoukian Division of Digestive Diseases, University of California, Los Angeles, Los Angeles, CA

[7]Pathology Department, University of California, Los Angeles, Los Angeles, CA

[8]Department of Human Genetics, University of California, Los Angeles, Los Angeles, CA

[9]Department of Surgery, University of California, Los Angeles, Los Angeles, CA

[10]Jonsson Comprehensive Cancer Center, University of California, Los Angeles, Los Angeles, CA

Supplemental Information

Table of Contents:

Materials and Methods……………………………………………………………………………………pg 3-11

Supplemental Tables……………………………………………………………………..………..………..pg 12-16

Supplemental Figures…………………………………………………………………....……………......pg 17-44

Materials and Methods

snRNAseq isolation.

Nuclei were isolated as previously described by our group.[1, 2] Briefly, liver tissues were cut into ~1 cm sections, immediately placed in cold digestion media, mechanically homogenized, and filtered. After centrifugation and resuspension in media, they were immediately taken to the UCLA Technology Center for Genomics and Bioinformatic where cell viability by nuclear staining and cell membrane integrity were evaluated using a 40X brightfield microscope. Samples that passed QC (n=18) had 3'GEX libraries generated at a target of 20K reads/nuclei, following which, the samples were sequenced on the NovaSeq X Plus 25B platform.

snRNAseq analyses.

We aligned the raw snRNAseq reads to the reference HG38 and performed UMI quantification using STARsolo.[3] CellBender[4] was used to account for ambient contamination and remove empty droplets. We employed four doublet detection tools and used the majority voting results to identify singlets, as suggested by Demuxafy.[5] QC and visualization were performed using the SCANPY module in Python.[6] Droplets with total reads exceeding 5 median absolute deviations, mitochondrial reads more than 10% of total inputs, or labeled as doublets were excluded from downstream analyses. We used a previously published harmonized liver reference panel consisting of three datasets,[7-10] and performed reference-based cell-type annotation using CellTypist[11] with the harmonized Human Cell Atlas liver data.[10] Subsequently, we conducted annotation-aware integration with CellHint[10] and included the patient identity as batch information. In addition to newly sequenced samples (n=10), we downloaded the snRNAseq dataset of HCC and adjacent non-tumor samples of 1 patient with MASLD-HCC (BS472) from the Gene Expression Omnibus (GEO) database (ID: GSE189175).[12]

Following data integration, we performed cell-type-specific differential gene expression (DEG) analysis using Memento.[13] We compared tumor versus adjacent non-tumor controls in a paired analysis (n=8), by including patient identity as a blocking factor. Additionally, we used only non-tumor samples to perform DEG analysis between cirrhotic and non-cirrhotic samples (n=7). The significance threshold was set at $FDR < 0.05$ for the

number of genes and cell-type groups tested after correction by multiple testing by FDR. We estimated gene-gene correlations in snRNAseq data by first aggregating raw read counts of nuclei into pseudo-bulk read counts by cell-type and sample. Kendall correlations were then calculated in 10 independent tumor samples based on bulk counts. To identify subpopulations within the HSC cell-type, we performed clustering within HSCs using the Leiden algorithm implemented in SCANPY.[14] We examined clustering at resolutions of 0.25, 0.5, and 1 and determined HSC subtypes using gene ranking analysis and marker expression.[15] Subclusters with fewer than 20 cells were excluded.

CosMx molecular imaging sample preparation.

Formalin-fixed paraffin-embedded (FFPE) human liver tissues were prepared for spatial imaging using the CosMx SMI platform following the manufacturer's Manual Slide Preparation for RNA Assays protocol. 5µm sections were mounted onto slides and immediately baked overnight at 37°C to improve tissue adherence prior to use or storage. Before the run, slides were baked overnight at 60°C in an oven propped at a 45° angle. Slides underwent sequential xylene and ethanol washes for deparaffinization, followed by heat-induced antigen retrieval for 20min at 100°C. After rinsing in water then ethanol, slides were air-dried for 30min at room temperature. Permeabilization was conducted using freshly prepared 3µg/mL Proteinase K digestion buffer for 30min at 40°C in a hybridization oven. Fiducial markers (0.001%) were applied for 5min, followed by PBS washing followed by post-fixation with 10% neutral buffered formalin for 1min and quenching with tris-glycine buffer. Tissue blocking was performed using sulfo-NHS-acetate.

In situ hybridization was carried out overnight at 37°C using the CosMx Human RNA 6K Discovery Panel. On the second day, unbound probes were removed by two rounds of stringent washes using a 1:1 mixture of 100% deionized formamide and 4x saline-sodium citrate buffer. Nuclear and cell segmentation markers were applied (DAPI, Hs CD298/B2M, and Hs PanCK/CD45). Prepared slides were assembled into flow cells using the Flow Cell Assembly Kit.

For imaging, liver tissues were processed using pre-bleaching profiles: Configuration-B (90s for adjacent non-tumors) and Configuration-C (60s for HCC), with liver-specific cell segmentation profile (Configuration-E).

Fields of view (FOVs) were selected based on initial immunostaining. Data acquisition was performed on the CosMx SMI instrument, and downstream image processing, transcript quantification, and spatial analysis were completed using the AtoMx Spatial Informatics Platform.[16]

CosMx analyses.

Initial cell segmentation was performed with CellPose[17] and was evaluated for artifacts such as abnormally large gaps between cells or boundaries that only captured nuclei. Re-segmentation was performed with different workflows and adjusting parameters such as cell dilation, nuclear diameter, cell diameter, minimum cell size, model, nuclei probability, cell flow threshold, and cell probability as needed until these artifacts were no longer present. QC filtering was performed with the AtoMx pipeline; FOVs were excluded from analysis if the mean transcript count per cell was not greater than 100 and individual cells were excluded based on default thresholds for transcript counts per cell, proportion of negative probe counts, cell complexity, and cell area. The AtoMx pipeline was also used to calculate cell number and size.

RNA expression was normalized using the LogNormalize method described in Seurat.[18] UMAP visualizations and spatial plots were generated with the DimPlot() and ImageDimPlot() functions.[18] Cell type assignment was performed with the InSituType algorithm[19] using reference data.[8] For BS414 and BS644, HCC tissue regions were segmented based on FOVs using the corresponding H&E stains as a reference. DEGs were calculated using the FindMarkers() function.[18]

Whole-genome sequencing (WGS) and data analysis.

Genomic DNA for WGS was extracted from 7µm sections of FFPE tissue using a Qiagen QIAamp DNA FFPE Tissue Kit as per the protocol. WGS was performed on the Illumina NovaSeq X Plus platform, generating 2x150bp paired-end reads, aiming for 7-8X coverage. We aligned WGS reads to the human genome reference, GRCh38, using BWA-mem2.[20] Duplicated reads were then marked using Picard v3.0 following GATK best practices.[21]

***Copy number variation (CNV) analysis*.**

CNV calls were conducted using the CNVkit.[22] Specifically, we used the "cnvkit batch" command to process all WGS data jointly with default parameters except for the following arguments: "-m wgs --drop-low-coverage." Briefly, we pooled 5 adjacent tissue samples from 4 patients to create a reference panel as controls. Each HCC sample was then compared against the reference to detect CNVs. CNVkit then applied bias correction on read depth to account for GC content and repeats. The log2 ratios of adjusted read depth were then calculated to determine the copy numbers of genomic segments.

Immunohistochemistry

FFPE tissues were cut into 5µm sections before adhering to microscope slides. The slides were heated to 70°C to melt the paraffin before rehydrating the tissues in incremental steps from 100% xylene, to varying percentages of ethanol, saline, and finally to 1x PBS pH 7.0. Slides were placed in 0.01 M sodium citrate buffer, pH 6.0 and heated in a steamer for 30 minutes for antigen retrieval. Once cool, the slides were incubated in 3% H_2O_2 solution for 5 minutes to quench any exogenous peroxidase activity. Tissues were washed in PBST solution (PBS with 0.1% Tween-20) 3 times for 5 minutes each wash before blocking in 5% goat serum for 1 hour at room temperature. Slides were washed with PBST again before incubating with either GLUL or HAMP 1° antibodies both at 1:200 dilution in 2% goat serum. Antibodies were left to incubate for 1h at room temperature before moving to 4°C overnight. The following day, the slides were washed with PBST prior to incubation with 2° antibodies – either biotinylated mouse for GLUL or biotinylated rabbit for HAMP, both at 1:500 in 2% goat serum. Antibodies were incubated for 1h at room temperature before washing with PBS and incubating with ABC peroxidase solution for 30 minutes at room temperature. Slides were washed with PBS again before the addition of DAB substrate. For all HAMP slides, adjacent and HCC tissues, DAB was left on the liver sections for 30 seconds before immersing in PBS to stop the reaction. For GLUL, all tissues were also left to incubate for 30 seconds besides adjacent tissues BS644 and BS818, which were left for 45 seconds

before immersing in PBS. Tissues were then counterstained with hematoxylin, dehydrated, and mounted with limonene mounting media before imaging on a ZEISS Axiocam 305 color at 10x.

Pathway analyses

Pathway analysis for snRNAseq and bulk RNAseq was performed using Reactome.[23, 24] For snRNAseq, DEGs for each cell type, either comparing non-cirrhosis vs. cirrhosis for the adjacent non-tumor samples (n=12) or adjacent vs. HCC for the paired samples (n=8), were filtered for FDR < 0.05 and then sorted for downregulated genes (difference in expression < 0) or upregulated genes (difference in expression > 0). For bulk RNAseq, adjusted p value < 0.05 was used as the significance filter and fold change > 2 was used to indicate upregulation and fold change < -2 for downregulation. Each gene set was imported into Reactome individually before evaluating output. Only pathways with an FDR < 0.05 were considered. Entities coverage was calculated by divided “entities found” by “entities total.”

Cell Culture

LX2 cells (SCC064, Sigma-Aldrich) and primary HSCs (donor 1: BioIVT, Westbury, NY; donors 2-4: LifeNet Health, Virginia Beach, VA) were maintained in monolayer culture at 37°C with 5% CO_2. Donor 1 had never been plated prior to pHSC isolation. Donors 2-3 had been plated prior to our experiments and donor 4 had been plated and passaged once prior to our experiments. All cells were maintained in Dulbecco’s modified Eagle medium which was supplemented with 10% fetal bovine serum (FBS) and 1x of Antibiotic-Antimycotic.

***Bulk RNA sequencing of 4 pHSC donors*.**

Donor 1 was obtained from BioIVT (XenoTech),[25] while donors 2-4 were obtained from LifeNet Health. After RNA isolation, RNA QC was assessed, and all 10 samples had an RNA integrity number of 10. All samples were sequenced via the UCLA TCGB on a Novaseq X plus 10B lane. We aligned reads to the GRCh38 human genome.[26] Adapter trimming and quality filtering were done with fastp[27] and all summarized with MultiQC.[28] All 10 samples had >99.8% reads that passed filtration. Quantification was conducted using

kallisto[29] and results were aggregated into one matrix using the tximport R-script.[30] DESeq2 was used for differential expression[31] and volcano plots were generated using ggplot2 and ggrepel in R.[32, 33]

Immunofluorescence

Cells were plated in Nunc™ glass bottom dishes (150682, Thermo Fisher Scientific) for microscopy. After the indicated treatment, cells were fixed with 4% paraformaldehyde for 30 minutes at room temperature. Cells were then washed with PBS and permeabilized with 0.4% Triton X-100 for 15 minutes at room temperature. Cells were then blocked with 2.5% BSA for 1 hour at room temperature. Anti-YAP antibody was used at 1:200 and added to cells overnight at 4°C. The following day, cell staining was achieved by incubating Alexa Fluor 488 (1:1,000) for 1 hour at room temperature prior to F-actin visualization using Alexa Fluor 546 phalloidin at 1:2,000 for 1 hour at room temperature. Nuclei were stained with Hoechst 33258 at 1:10,000 for 5 minutes before mounting with AquaSlip (MMC0619, American Mastertech Scientific). Cells were either imaged using EVOS 5000 FL Cell Imaging System (Thermo-Fisher Scientific) at 40x magnification or Leica THUNDER widefield fluorescence microscope at 63x oil immersion (NA=1.4 63x lens). Image collection on the Leica THUNDER widefield fluorescence microscope was performed at the Advanced Light Microscopy/Spectroscopy Laboratory and Leica Microsystems Center of Excellence at the California NanoSystems Institute at UCLA (RRID:SCR_022789) with funding support from NIH Shared Instrumentation Grant S10OD025017 and NSF Major Research Instrumentation grant CHE-0722519. To quantify YAP localization, ratio measurements of nuclear to total cellular YAP from at least 20 images were collected using ImageJ software (National Institutes of Health (NIH)).

RNA Purification, cDNA Synthesis, and RT-qPCR

LX2 cells and pHSCs were treated as indicated before the direct addition of TRIzol (Invitrogen) and subsequent RNA isolation using Direct-zol (R2052, Zymo Research, Irvine, CA). cDNA was synthesized using iScript Reverse Transcriptase Supermix (no. 1708841, Bio-Rad, Hercules, CA) according to the manufacturer's protocol. RNA was quantified using a Nanodrop 2000 (Thermo-Fisher Scientific). Relative quantitate RNA RT-

qPCR was subsequently conducted using iTaq Universal SYBR Green Supermix (no. 1725121, Bio-Rad) in the 384 Touch Bio-Rad Thermocycler. *36B4* was used as an internal control and used to normalize the gene expression data. The list of primers is provided in **Table S7.**

Western Blots

LX2 cells were grown and washed in 6-well tissue culture plates with PBS and treated overnight in 1μM cerivastatin or 20μM atorvastatin for 24 hours in 0.5% FBS media. The cells were then lysed in RIPA buffer Santa Cruz Biotechnology (sc-24948, Dallas, TX), pelleted, and the supernatant removed. SDS-PAGE sample buffer was used to resuspend the pellet, followed by running an SDS-PAGE on 4%–20% gels, and transferred to immobilon P membranes (Millipore, Burlington, MA). The membranes were blocked in 5% nonfat dried milk in PBS and incubated overnight in 1:1,000 anti-YAP antibody. Secondary anti-rabbit, HPR-linked antibody was used at 1:10,000 (7074, Cell Signaling Technology, Danvers, MA). Antibody bands were visualized by enhanced chemiluminescence (ECL) detection with horseradish peroxidase (HRP)-conjugated anti-rabbit antibody (7074S, Cell Signaling Technology), imaged using Bio-Rad ChemiDoc Touch Imaging System, and quantified using Image Lab (V6.1).

Quantitation of geranylgeranyl pyrophosphate (GGPP).

GGPP was measured by Ultra High-Performance Liquid Chromatography-tandem mass spectrometry (UPLC-MS/MS) on a platform comprising Waters Xevo TQA triple quadrupole mass spectrometer integrated with a Acquity Premier LC. GGPP was extracted from the cell lysates by mixing with chilled methanol for protein precipitation. After centrifugation, the supernatant was evaporated under a nitrogen stream and suspended in 20% acetonitrile for analysis. Chromatographic separation was done on a Waters Acquity UPLC BEH C18 column (1.7μm, 2.1 X 50mm) maintained at 40°C. The initial conditions were 90% Solvent A (10mM ammonium bicarbonate containing 0.5% ammonium hydroxide) and 10% Solvent B (90% acetonitrile with 0.5% ammonium hydroxide) at 0.4mL/min. Solvent B was increased linearly to 40% by 7min, to 100% by 8min, held for 1min and back to initial conditions by 9.1min with a total run time of 10.5min. Negative ESI-MS/MS mass spectrometry under MRM mode was performed using following transitions: 448.97>78.90 (quantifier) and

448.97>158.87 (qualifier). The GGPP concentration was measured by comparing integrated peak areas against known amounts of GGPP using Targetlynx V4.2. The limit of quantitation of the assay is 0.05ng/mL. The mean intra-and inter-assay imprecision was 3.5% and 4.43% respectively.

Cell Viability Assay

Cell viability measurement used to quantify atorvastatin tolerance were conducted in a 96-well plate and collected using Cell counting kit-8 (GLPBIO, Montclair, CA). After treatment, the reagent was added to the cells and incubated for 3-hours in the incubator. Prior to the absorbance measurement, the plate was rocked for 3 minutes to ensure an even distribution. Absorbance readings were collected at 450nm and the viability was quantified relative to a control.

Statistical Analysis

Patient baseline characteristics were presented as total quantity, mean with standard deviation (SD) or median with interquartile ranges. Graphs are presented as means or median ± SD. Experimental conditions are compared by the Mann–Whitney *U* test, one-way ANOVA, or Student's *t* test using Prism 10.4.0 (GraphPad Software, Inc., San Diego, CA). Statistical analysis used and p-values are indicated in the figure legends when applicable. $P<0.05$ was considered statistically significant.

SUPPLEMENTAL TABLES

Table S1: DEGs of subcluster 1, 2, and 3 v all others determined by snRNAseq (excel file).

Table S2: DEGs determined by snRNAseq by cell-type by cirrhosis and tumor status (excel file).

Table S3: Reactome pathways analysis of snRNAseq DEGs (excel file)

Table S4: Tissue cell statistics: FOV number, cell count, and average cell size.

Tissue/Fibrosis Staging	Status	Number of FOVs	Total cell count	HSCs count	Average HSCs/FOV	Average cell size (μm^2)	Standard deviation cell size (μm^2)
BS217- F0	normal	124	131,828	4,042	32.60	238.93	114.92
BS414- F2	adjacent	124	123,503	4,285	34.56	251.39	135.74
BS414- F2	HCC (not sectioned)	210	237,828	5,848			
	Section A	53	54,617	2,041	38.51	218.90	112.98
	Section B	40	37,490	2,815	70.38	184.97	96.15
	Section C	117	145,721	992	8.48	163.89	78.22
BS460- F2-3	adjacent	124	152,745	8,433	68.01	201.69	115.25
BS460- F2-3	HCC	236	262,512	2,429	10.29	183.42	95.69
BS551- F0	HCC	9	14,462	3	0.33	142.00	63.10
BS630- F4	HCC	137	99,659	11,977	87.42	195.46	118.50
BS644- F4	Adjacent	104	81,334	9,408	90.46	310.28	204.18
BS644- F4	HCC (not sectioned)	57	56,961	2,749			
	Section B	11	10,176	2,146	195.09	166.11	79.98
	Section C	46	46,785	603	13.11	171.02	97.75
BS811- F4	Adjacent	35	27,039	1,270	36.29	319.47	184.33
BS818- F4	HCC	151	89,388	1,749	11.58	233.15	172.01

Table S5: Clinical phenotype of pHSC donors

**Donor number**	**Age**	**Sex***	**Race**	**BMI**	**Fibrosis or history of liver disease**
1	63	F	Caucasian	39	No known liver disease
2	57	F	Caucasian	61	No liver disease, F=0, NAS**=3
3	24	F	Caucasian	33	No liver disease, F=0, NAS=0
4	34	F	Hispanic	26	No liver disease, F=0, NAS=3

*First donor was randomly a female from BioIVT. Donors 2-4 from LifeNet were therefore selected to be female to decrease sex-specific differences. **NAS = NAFLD activity score.

Table S6: Downregulated genes with statin treatment identified by bulk RNA sequencing of pHSCs.

Downregulated by cerivastatin only	Downregulated by atorvastatin only	Downregulated by both statins
ALPK2	*ACTBL2*	*ANKRD1*
ANLN	*ADGRL3*	*APLN*
ANOS1	*AJUBA*	*APOBEC3B*
ANXA8	*ALDH1A1*	*ARHGEF39*
ANXA8L1	*AMOTL2*	*ASPM*
AP1S3	*ARNT2*	*AURKB*
APCDD1	*BCL2A1*	*B3GALT2*
ARAP2	*BDNF*	*BEX1*
ASF1B	*BRINP1*	*BUB1B*
BIRC5	*CARMN*	*CCN1*
BMX	*CCBE1*	*CCN2*
C3AR1	*CCN4*	*CCNA1*
CALB2	*CEMIP*	*CDC20*
CAP2	*CITED4*	*CDC25C*
CASP4LP	*CNN2*	*CDCA3*
CASS4	*CTHRC1*	*CDK1*
CCL2	*CXADR*	*CENPA*
CCNB2	*DAAM2*	*CKAP2L*
CCNE2	*DNAAF4-CCPG1*	*COLEC10*
CD200	*EDN1*	*DIRAS3*
CDCA8	*ESPL1*	*DLGAP5*
CDKN3	*FLRT3*	*DTL*
CENPF	*GCSHP5*	*ERCC6L*
CENPM	*GDF5*	*EVA1A*
CEP55	*HLA-H*	*FAM111B*
COPG2IT1	*IFIT1*	*FAM83D*
CSF2RB	*IL1RL1*	*FGF1*
CXCR4	*INHBA*	*FMO2*
DAB1	*IRAG1*	*GAS6-AS1*
DDIAS	*KCNJ8*	*GREM1*
DEPDC1	*KRT18*	*GTSE1*
DYNLT5	*LINC00968*	*HJURP*
E2F8	*LINC01118*	*HMMR*
ESCO2	*LIPG*	*INMT*
FGFR2	*LMOD1*	*IQGAP3*
FMO6P	*MPP4*	*KIF14*
GINS2	*NPR3*	*KIF18B*
GPR65	*NUAK2*	*KIF20A*
HEYL	*PPFIA4*	*KIF26B*
IL6	*PRXL2A*	*KIF2C*
KCNAB1	*PWWP3B*	*LINC02709*
KCNQ5	*RASL10B*	*LMNB1*

KCNS1	*RGCC*	*MARCHF4*
KIF23	*RGS18*	*MCM10*
KIF4A	*RNF150*	*MDFI*
KNL1	*SAMSN1*	*MEST*
LINC01235	*SFXN2*	*MKI67*
LZTS1	*SLC16A12*	*MYB*
MCOLN2	*SPHK1*	*MYBL2*
MGARP	*STAMBPL1*	*NCAPH*
MGAT4C	*STC1*	*NEIL3*
MIR503HG	*TGM2*	*NEK2*
MOV10L1	*TK1*	*NRXN3*
NIPAL1	*TRIP13*	*NUF2*
NRARP	*TROAP*	*OMG*
NUSAP1	*VDR*	*PBK*
OIP5		*PIMREG*
OXTR		*PLK1*
PCLO		*PTX3*
PKMYT1		*RFLNA*
SFTA1P		*RRM2*
SGO1		*SAPCD2*
SH2D5		*SHCBP1*
SLC1A7		*SIK1*
SMIM39		*SKA1*
SPAG5		*SLC38A4*
SSTR1		*SPC25*
SYT16		*THBS1*
TEDC2		*TICRR*
TMEM88		*TNFRSF11B*
TMPO-AS1		*TNFRSF12A*
TMSB4XP6		*TOP2A*
TSPOAP1		*TRIL*
UHRF1		*TTK*
VPS9D1-AS1		*TYRP1*
		UBE2C
		VCAM1

Table S7: Upregulated genes with statin treatment identified by bulk RNA sequencing of pHSCs.

Upregulated by cerivastatin only	Upregulated by atorvastatin only	Upregulated by both statins
7SK	*ACE*	*ADGRD1*
ATF3	*ADAMTS5*	*ADGRG2*
C12orf56	*AHNAK2*	*AQP3*
CDH23	*ALKAL2*	*C11orf96*
CLDN4	*C2CD4B*	*C5AR2*
CYP3A4	*CCN3*	*CD24*
DIRAS1	*CD55*	*CKB*
DLX3	*CD9*	*CRIP1*
DNAAF3-AS1	*CFD*	*CSPG4*
GPD1	*CREB5*	*DUSP8*
HAS1	*DRAXIN*	*FABP3*
IGSF9B	*ELL3*	*GJB2*
KCNN4	*EMX2*	*HSPA12B*
KRT14	*EPB41L3*	*ITGA9*
LILRB3	*FGD5*	*KLF2*
LRRN4CL	*GPAT3*	*KLF4*
MTUS2	*H19*	*LSP1*
MYBPH	*HSPA1A*	*MALL*
NTN4	*HTRA3*	*MMP28*
PKP3	*HYAL1*	*NGEF*
RADIL	*IQCN*	*NOVA2*
RPTN	*ITGA6*	*PAQR5*
SHOX2	*JUN*	*PTPRH*
TM4SF19	*KAZALD1*	*RHOB*
TMOD1	*KLF8*	*RTN4RL1*
VWA7	*KLK1*	*S100A4*
WNT2	*LINC00520*	*SCARA5*
ZNF385A	*LINC01133*	*SDK2*
	MIAT	*SYNE3*
	MN1	*SYT7*
	MYO1F	*THBD*
	NMRAL2P	*TIMP3*
	P3R3URF-PIK3R3	*TNNT1*
	PALM	*TNXB*
	PCDH1	*WFDC21P*
	PIK3C2B	WNT9A
	PLIN4	
	PRG4	
	SMANTIS	
	TEK	
	VSIG10L	
	VWF	

	ZNF731P	

Table S8: Primer sequences used for qPCR.

**Primer**	**Sequence**
36B4-F	CCACGCTGCTGAACATGCT
36B4-R	TCGAACACCTGCTGGATGAC
YAP-F	AGTGGACTAAGCATGAGCAG
YAP-R	TGTTCATTCCATCTCCTTCC
TAZ-F	GGCTGGGAGATGACCTTCAC
TAZ-R	CTGAGTGGGGTGGTTCTGCT
CCN2-F	AGGAGTGGGTGTGTGACGA
CCN2-R	CCAGGCAGTGGCTCTAATC
CCN1-F	AGCCTCGCATCCTATACAACC
CCN1-R	TTCTTTCACAAGGCGGCACTC
HMGCS1-F	GCACAGTACTCACCTCAGCA
HMGCS1-R	CCCGGTGTAGCATCTTGTGT
LDLR-F	GCCTCTGAAATGCCTCTTCT
LDLR-R	CCCAGAAGCCACTCATACATAC
ACTA2-F	AGGCACCCCTGAACCCCAA
ACTA2-R	CAGCACCGCCTGGATAGCC
Col1A-F	GGCTTCCCTGGTCTTCCTGG
Col1A-R	CCAGGGGGTCCAGCCAAT

SUPPLEMENTAL FIGURES

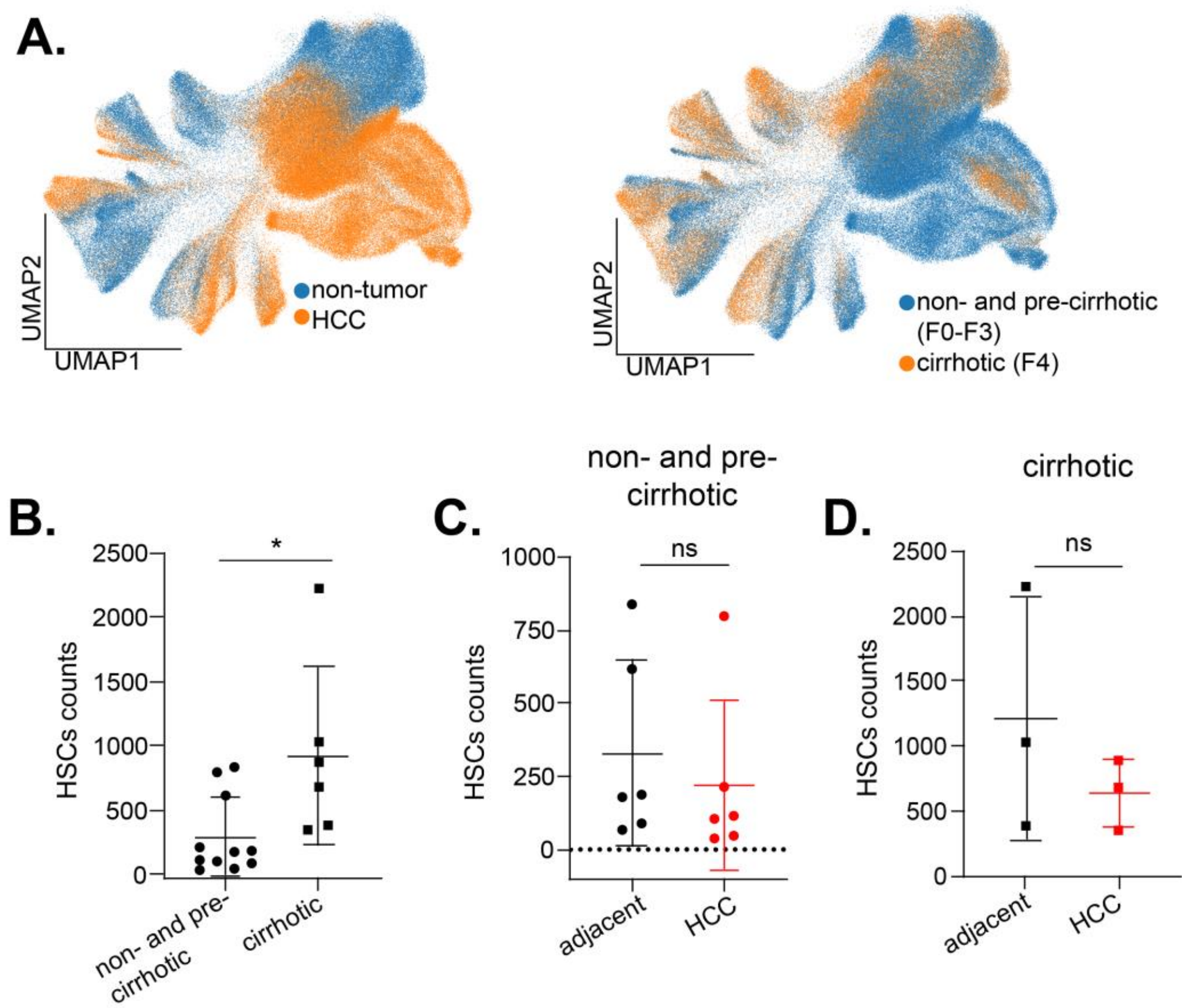

Figure S1: A) UMAP plots by tumor (left) and cirrhosis status (right).HSCs counts from snRNAseq data of **B)** non- and pre-cirrhotic vs. cirrhotic samples. Cirrhotic samples had a higher number of HSCs (mean 925 $\pm$ 690), compared to non- and pre-cirrhotic samples (293 $\pm$ 303, *P=0.0103) by Mann-Whitney U-test. Separating non- and pre-cirrhotic **(C)** and cirrhotic **(D)** into adjacent and HCC showed no significant difference with HSCs counts in HCC-tumor tissues compared to the adjacent non-tumor controls for each group.

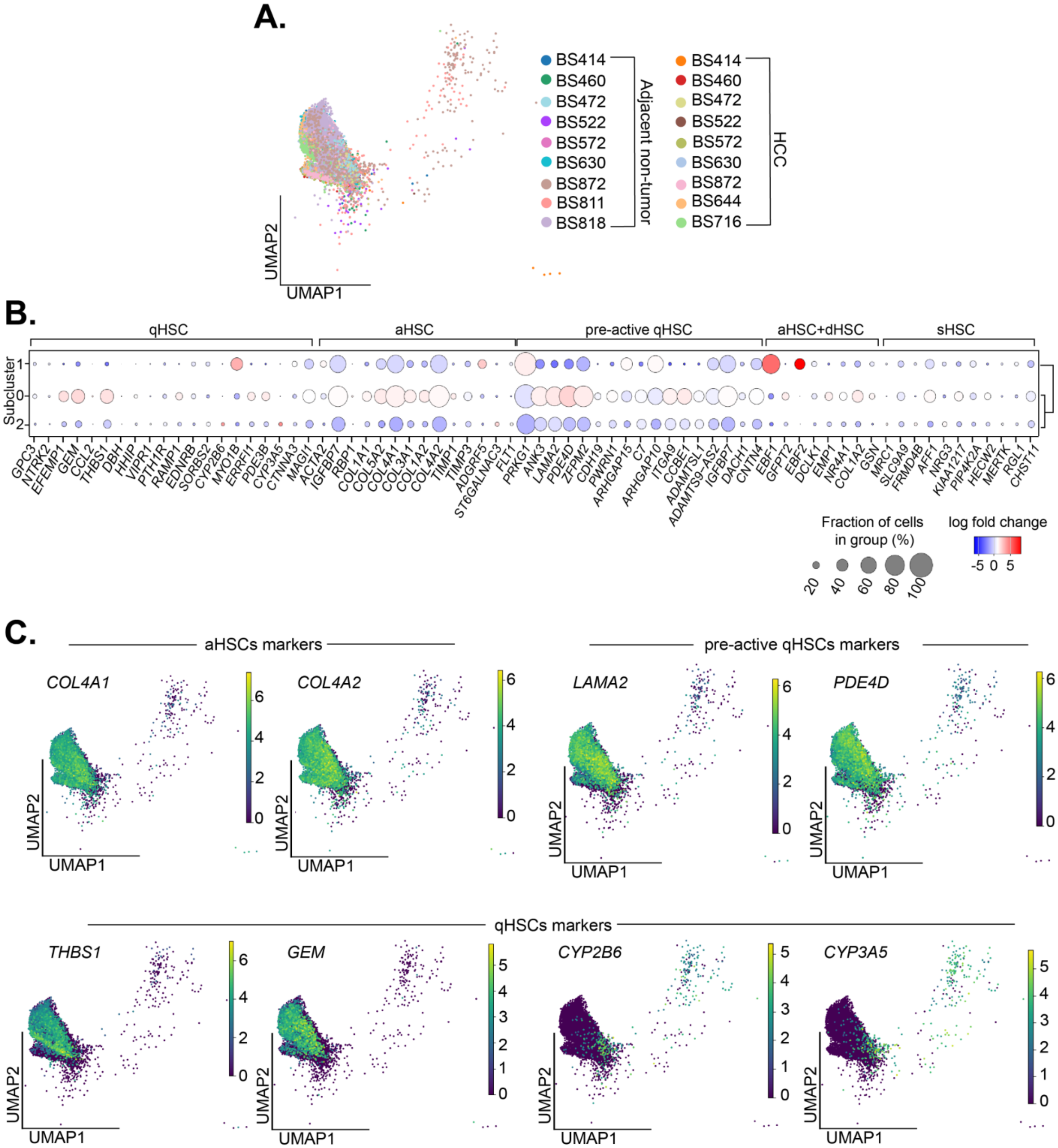

Figure S2: A) UMAP subclustering of HSCs shown by patient contribution from both non-tumor and tumor tissues. **B)** Dotplot of select quiescent and activated HSC genes show that cluster 1 is predominantly composed of activated + deactivated HSC signature genes. **C)** Select marker genes for aHSC, pre-active qHSCs, and qHSCs help resolve clusters 0, 1, and 2 phenotype.

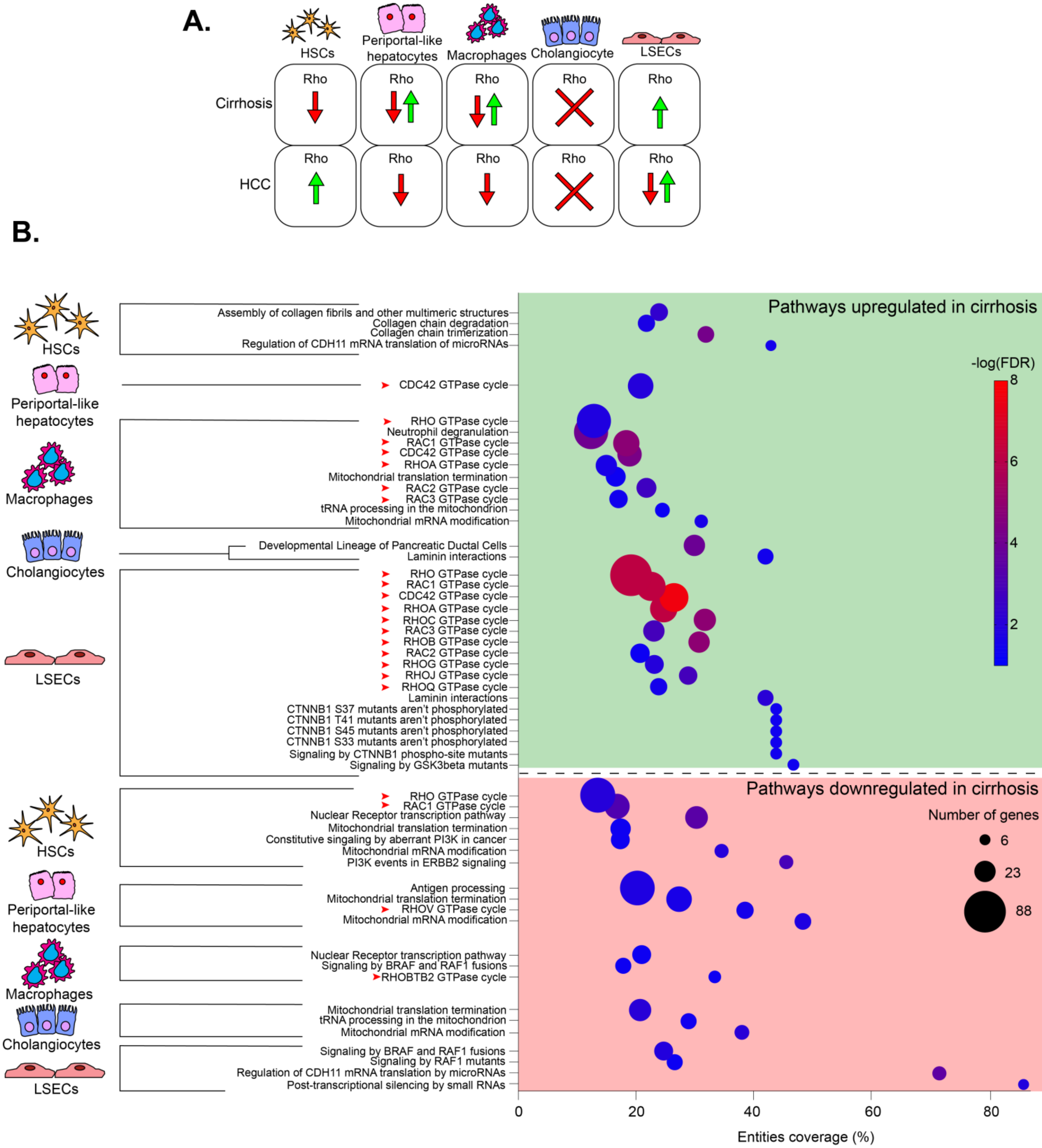

Figure S3: A) Schematic summary of Rho GTPase pathways for cell-types by tumor and cirrhosis status. **B)** Pathway analyses of DEGs by Reactome for MASLD-HCCs by cirrhosis status.

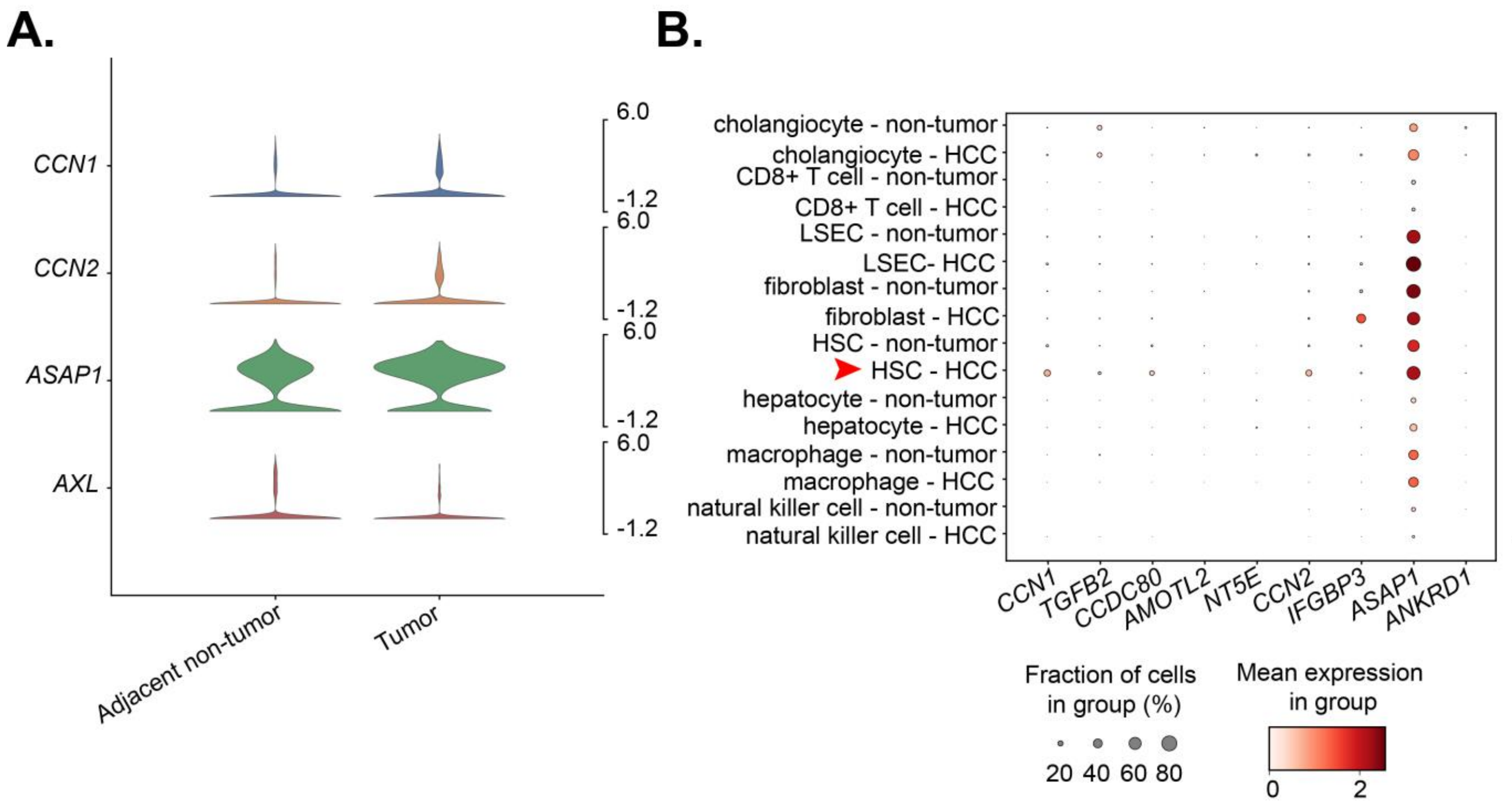

Figure S4: A) DEGs of select YAP-effectors genes (*CCN1, CCN2, ASAP1,* and *AXL*) between adjacent non-tumor and tumor (n=7 paired samples only) for all cell types. **B)** YAP-effector genes have a higher expression in HCCs,compared to adjacent non-tumor controls, predominantly in HSCs (red arrow) (n=18 samples). *FDR* < 2.60 E-06 for *CCN1, CCN2,* and *ASAP1* in HSC-HCC.

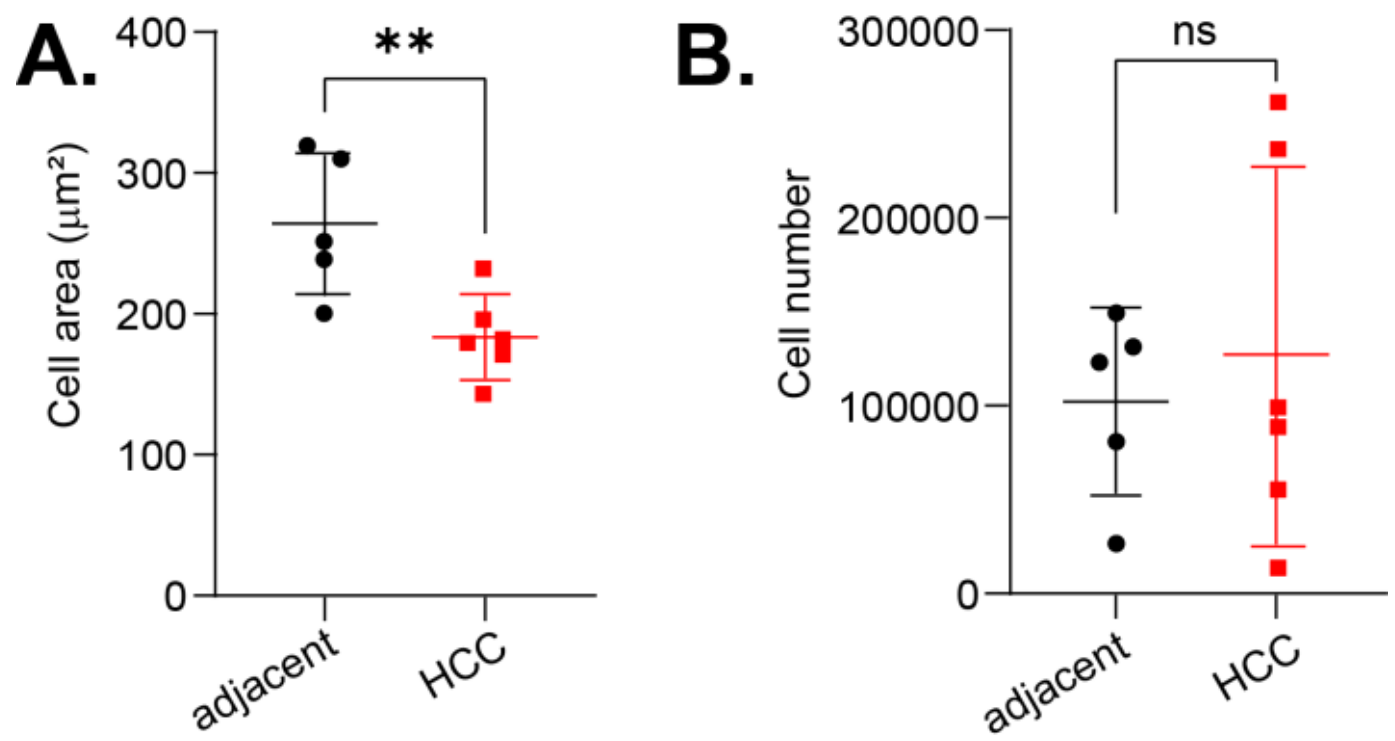

Figure S5: Between adjacent non-tumors and HCCs, HCC cells were smaller **(A)** (***P*= 0.0087) and trended towards having a greater number of cells overall **(B)**.

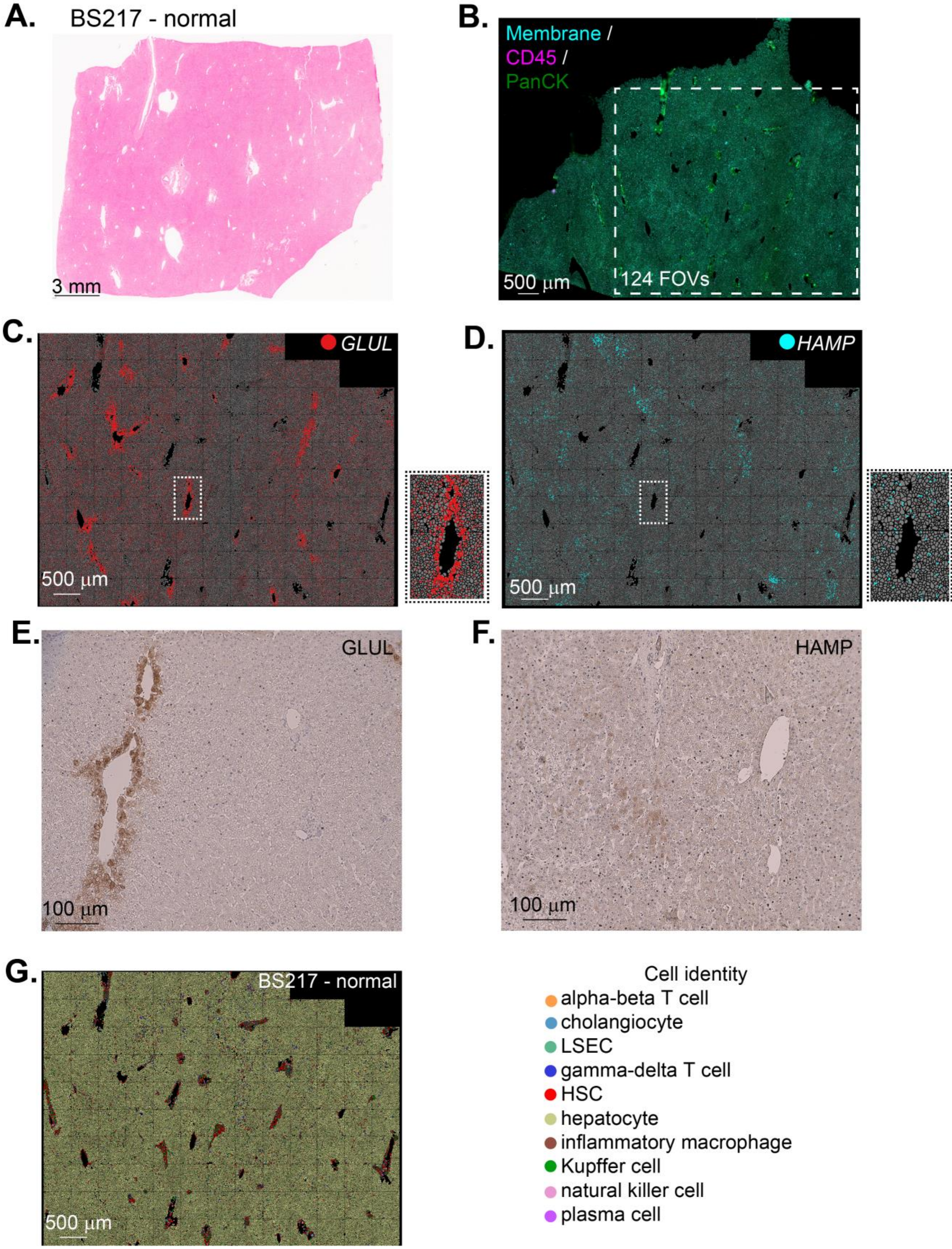
A.
BS217 - normal
3 mm
B.
Membrane /
CD45 /
PanCK
500 μm
124 FOVs
C.
GLUL
500 μm
D.
HAMP
500 μm
E.
GLUL
100 μm
F.
HAMP
100 μm
G.
BS217 - normal
500 μm
Cell identity
alpha-beta T cell
cholangiocyte
LSEC
gamma-delta T cell
HSC
hepatocyte
inflammatory macrophage
Kupffer cell
natural killer cell
plasma cell

Figure S6: Workflow of CosMx stRNAseq demonstrating **A)** H&E, **B)** area sequenced by FOVs (n=124). **C)** *GLUL* (glutamine synthetase) and **D)** *HAMP* (hepcidin antimicrobial peptide) gene expression by CosMx and **E-F)** IHC demonstrate central vein hepatocyte zonation by GS in zone 3 and HAMP in zones 1-2 in normal liver. **G)** Cell-type referencing of BS217.

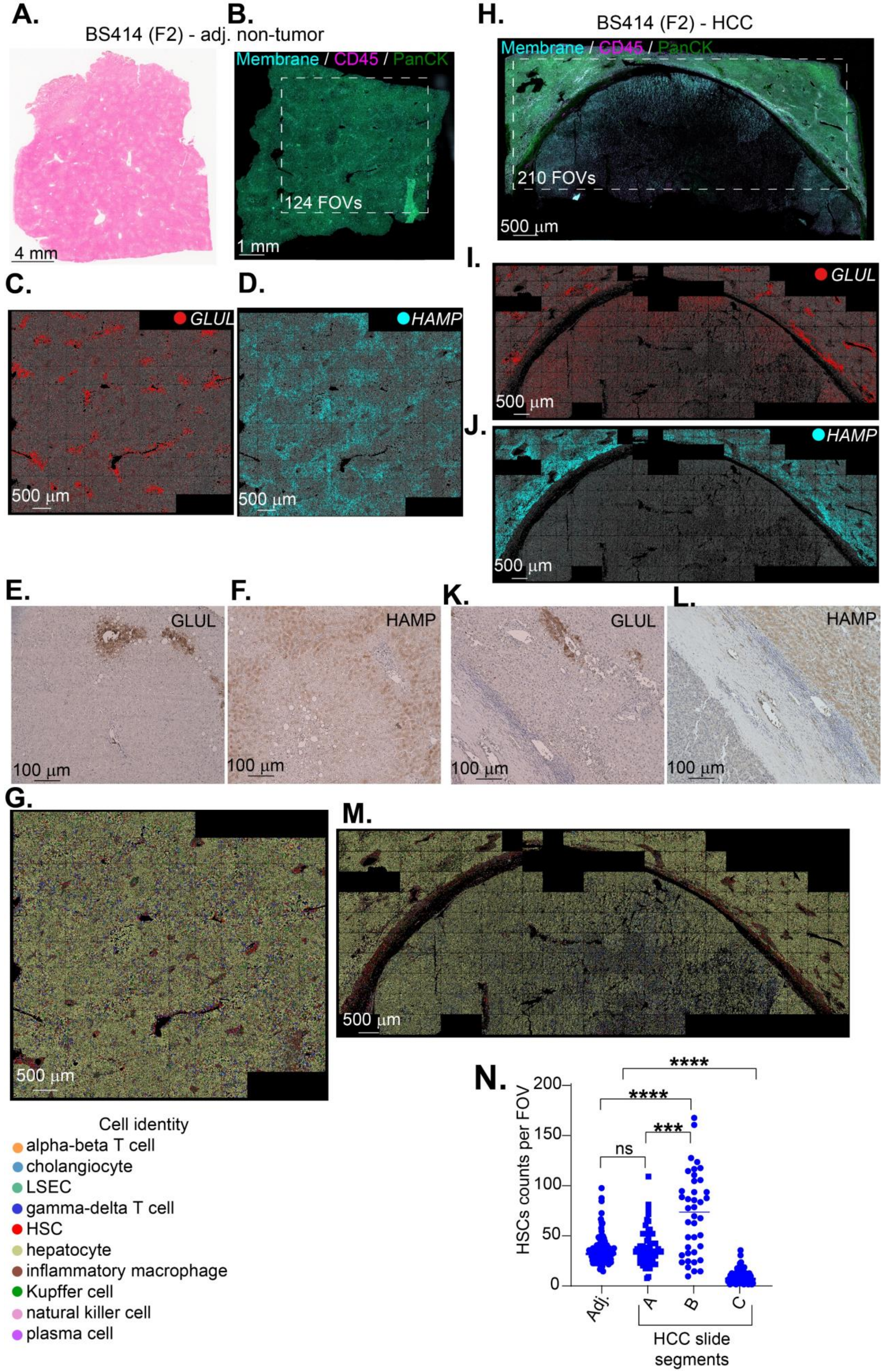
A.
BS414 (F2) - adj. non-tumor
4 mm
B.
Membrane / CD45 / PanCK
124 FOVs
1 mm
H.
BS414 (F2) - HCC
Membrane / CD45 / PanCK
210 FOVs
500 μm
C.
GLUL
500 μm
D.
HAMP
500 μm
I.
GLUL
500 μm
J.
HAMP
500 μm
E.
GLUL
100 μm
F.
HAMP
100 μm
K.
GLUL
100 μm
L.
HAMP
100 μm
G.
500 μm
M.
500 μm
Cell identity
alpha-beta T cell
cholangiocyte
LSEC
gamma-delta T cell
HSC
hepatocyte
inflammatory macrophage
Kupffer cell
natural killer cell
plasma cell
N.
HSCs counts per FOV
200
150
100
50
0
ns
***
****
****
Adj.
A
B
C
HCC slide segments

Figure S7: A) H&E, **B)** area sequenced by FOVs (n=124). **C)** *GLUL* and **D)** *HAMP* gene expression by CosMx and **E-F)** IHC demonstrate central vein hepatocyte zonation by GS in zone 3 and HAMP in zones 1-2 in adjacent BS414. **G)** Cell-type referencing of BS414. HCC – tumor BS414 tissue shown in **(H-M). N)** Quantification of HSCs per FOV in BS414 by segment.

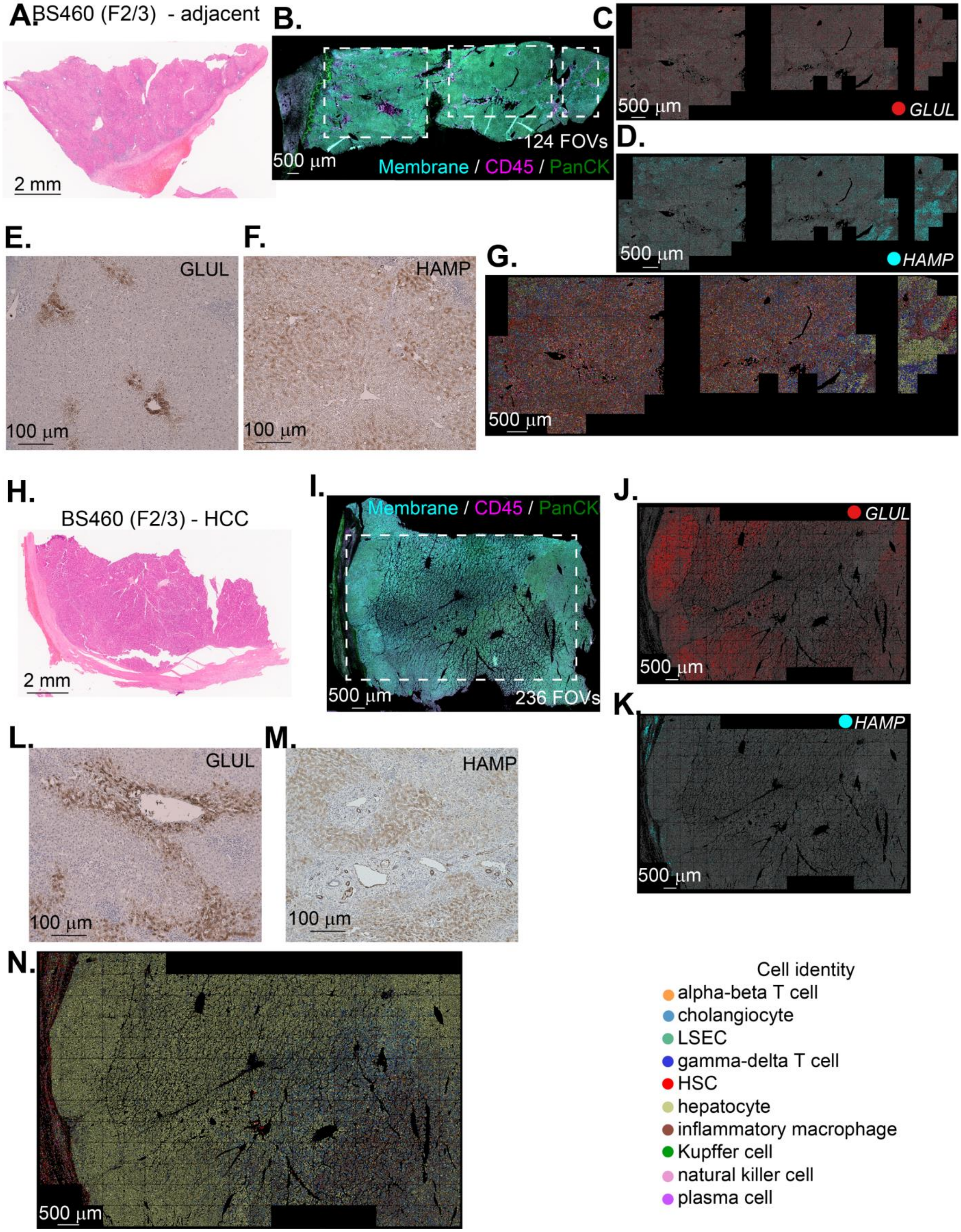
A. BS460 (F2/3) - adjacent
2 mm
B.
124 FOVs
500 µm
Membrane / CD45 / PanCK
C
500 µm
GLUL
D.
500 µm
HAMP
E.
GLUL
100 µm
F.
HAMP
100 µm
G.
500 µm
H.
BS460 (F2/3) - HCC
2 mm
I.
Membrane / CD45 / PanCK
500 µm
236 FOVs
J.
GLUL
500 µm
K.
HAMP
500 µm
L.
GLUL
100 µm
M.
HAMP
100 µm
N.
500 µm
Cell identity
alpha-beta T cell
cholangiocyte
LSEC
gamma-delta T cell
HSC
hepatocyte
inflammatory macrophage
Kupffer cell
natural killer cell
plasma cell

Figure S8: A) H&E, **B)** area sequenced by FOVs (n=124). **C)** *GLUL* (glutamine synthetase) and **D)** *HAMP* (hepcidin antimicrobial peptide) gene expression by CosMx and **E-F)** IHC demonstrate central vein hepatocyte zonation by GS in zone 3 and HAMP in zones 1-2 in adjacent tissue **G)** Cell-type referencing of BS460. HCC – tumor BS460 tissue shown in **(H-N).**

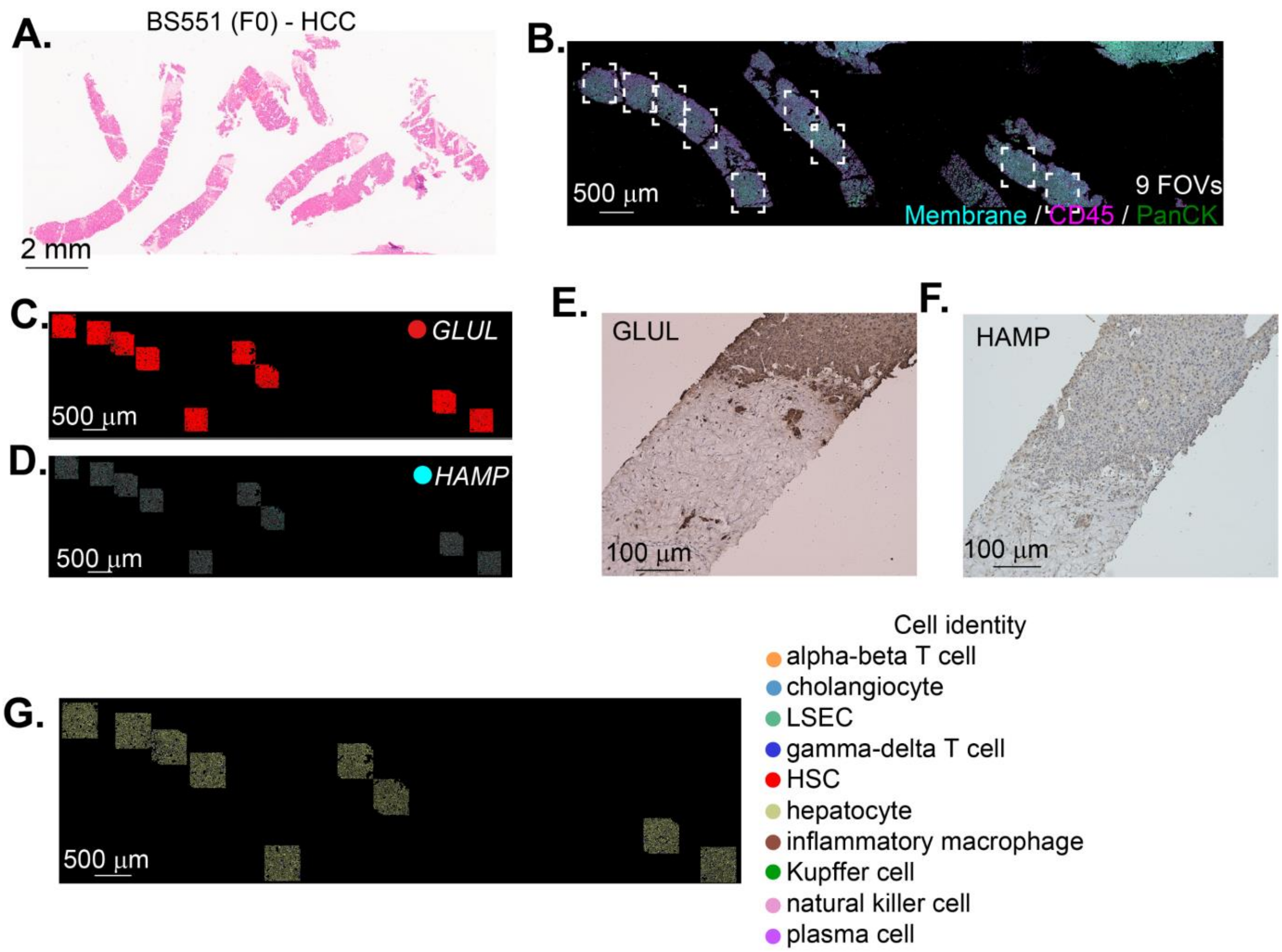

Figure S9: A) H&E, **B)** area sequenced by FOVs (n=9). **C)** *GLUL* and **D)** *HAMP* expression by CosMx and **E-F)** IHC demonstrate central vein hepatocyte zonation by GS in zone 3 and HAMP in zones 1-2 in BS551-HCC. **G)** Cell type referencing of BS551 HCC – tumor (core biopsy) tissue.

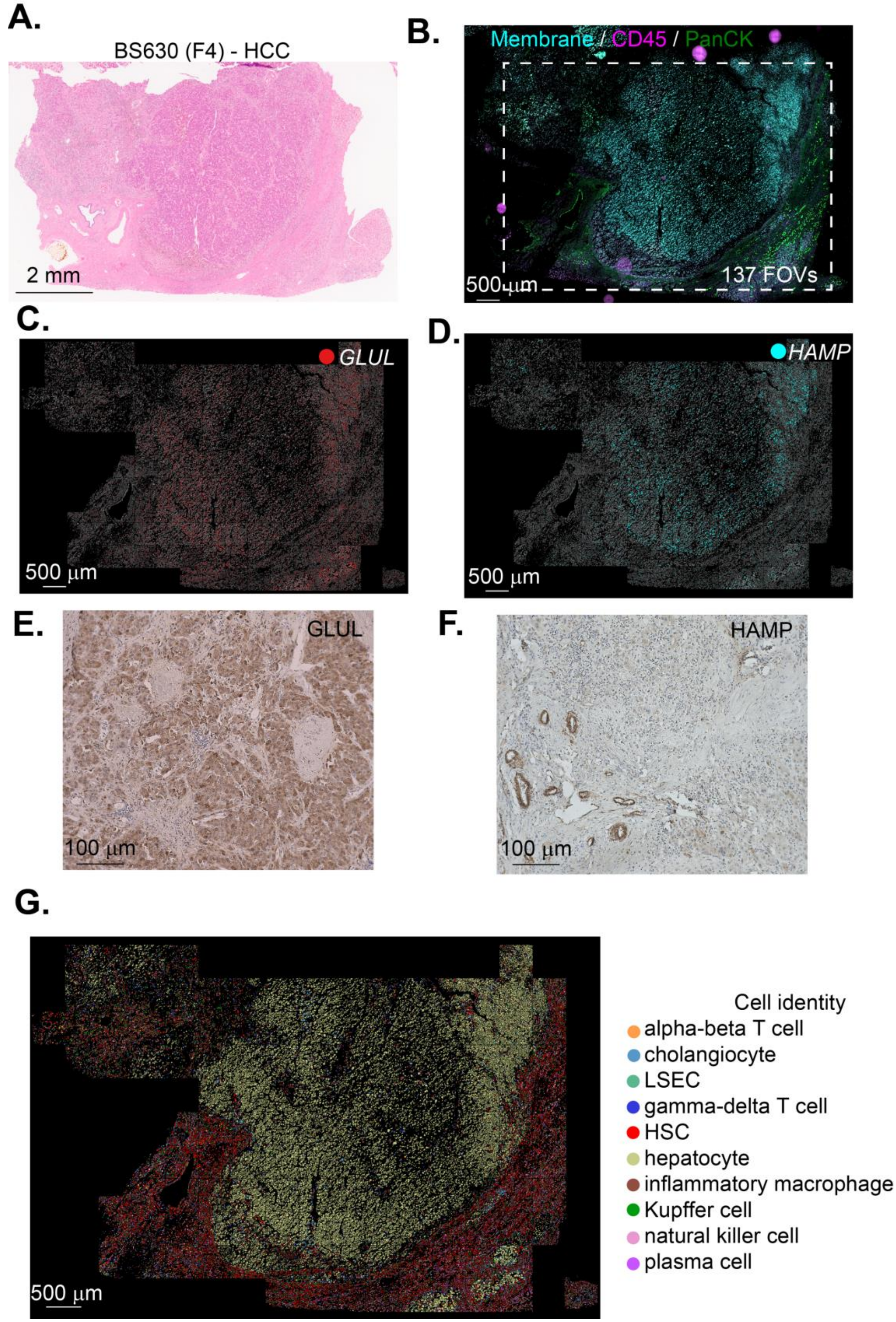

A.
BS630 (F4) - HCC
2 mm
B.
Membrane / CD45 / PanCK
137 FOVs
500 μm
C.
GLUL
500 μm
D.
HAMP
500 μm
E.
GLUL
100 μm
F.
HAMP
100 μm
G.
500 μm
Cell identity
alpha-beta T cell
cholangiocyte
LSEC
gamma-delta T cell
HSC
hepatocyte
inflammatory macrophage
Kupffer cell
natural killer cell
plasma cell

Figure S10: A) H&E, **B)** area sequenced by FOVs (n=137). **C)** *GLUL* and **D)** *HAMP* expression by CosMx and **E-F)** IHC demonstrate central vein hepatocyte zonation by GS in zone 3 and HAMP in zones 1-2 in BS630 HCC tissue. **G)** Cell-type referencing of BS630 – HCC.

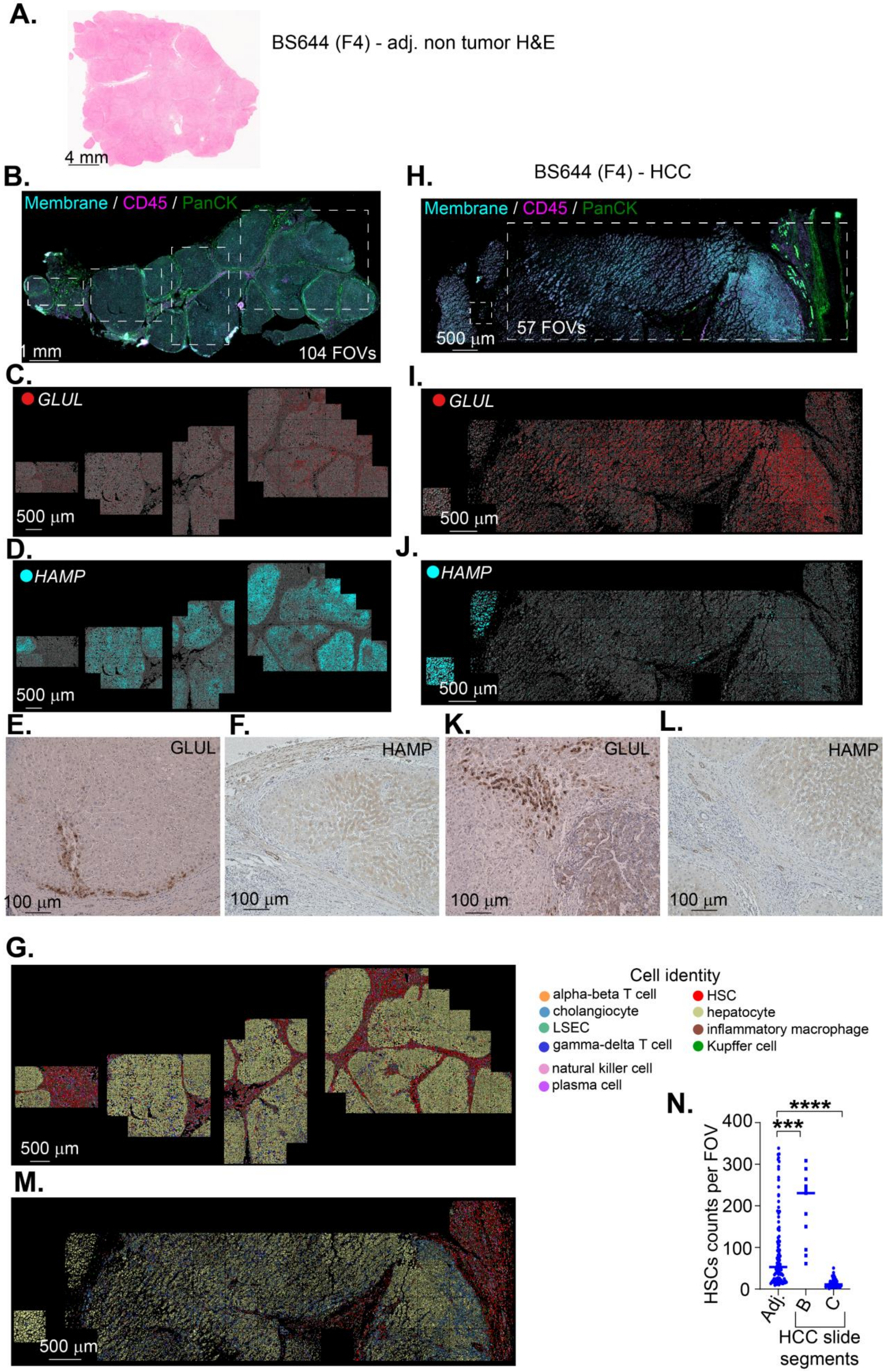
A.
BS644 (F4) - adj. non tumor H&E
4 mm
B.
Membrane / CD45 / PanCK
1 mm
104 FOVs
H.
BS644 (F4) - HCC
Membrane / CD45 / PanCK
500 µm
57 FOVs
C.
GLUL
500 µm
I.
GLUL
500 µm
D.
HAMP
500 µm
J.
HAMP
500 µm
E.
GLUL
100 µm
F.
HAMP
100 µm
K.
GLUL
100 µm
L.
HAMP
100 µm
G.
500 µm
Cell identity
alpha-beta T cell
cholangiocyte
LSEC
gamma-delta T cell
natural killer cell
plasma cell
HSC
hepatocyte
inflammatory macrophage
Kupffer cell
M.
500 µm
N.
HSCs counts per FOV
400
300
200
100
0
Adj.
B
C
HCC slide segments
***
****

Figure S11: A) H&E, **B)** area sequenced by FOVs (n=104). **C)** *GLUL* and **D)** *HAMP* gene expression by CosMx and **E-F)** IHC demonstrate central vein hepatocyte zonation by GS in zone 3 and HAMP in zones 1-2 in BS644 adjacent tissue. **G)** Cell-type referencing of BS644. HCC – tumor BS644 tissue shown in **(H-M). N)** Quantification of HSCs per FOV in BS644 by segment.

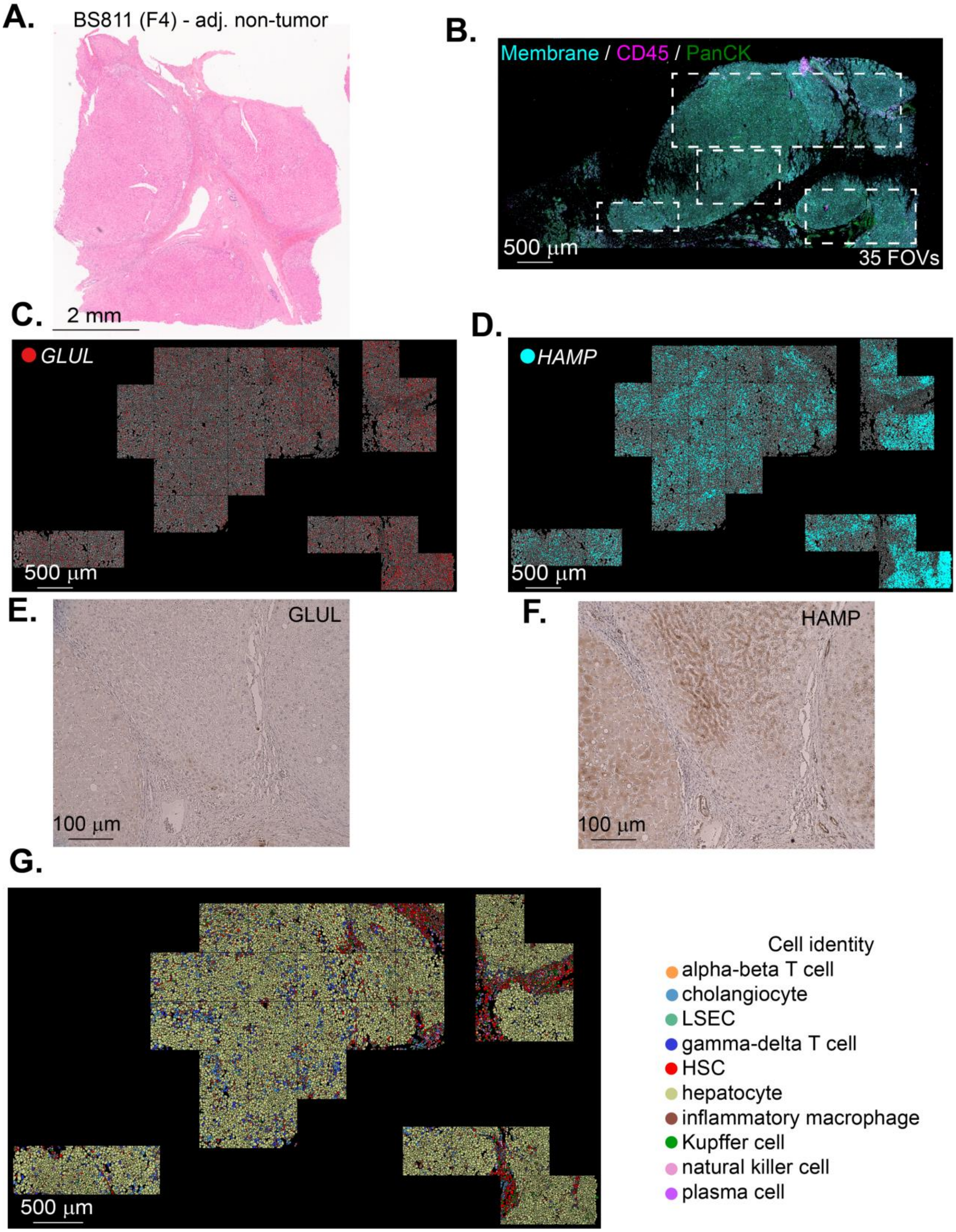

Figure S12: A) H&E, **B)** area sequenced by FOVs (n=35). **C)** *GLUL* and **D)** *HAMP* expression by CosMx and **E-F)** IHC demonstrate central vein hepatocyte zonation by GS in zone 3 and HAMP in zones 1-2 in BS811 adjacent tissue. **G)** Cell-type referencing of BS811 – adjacent.

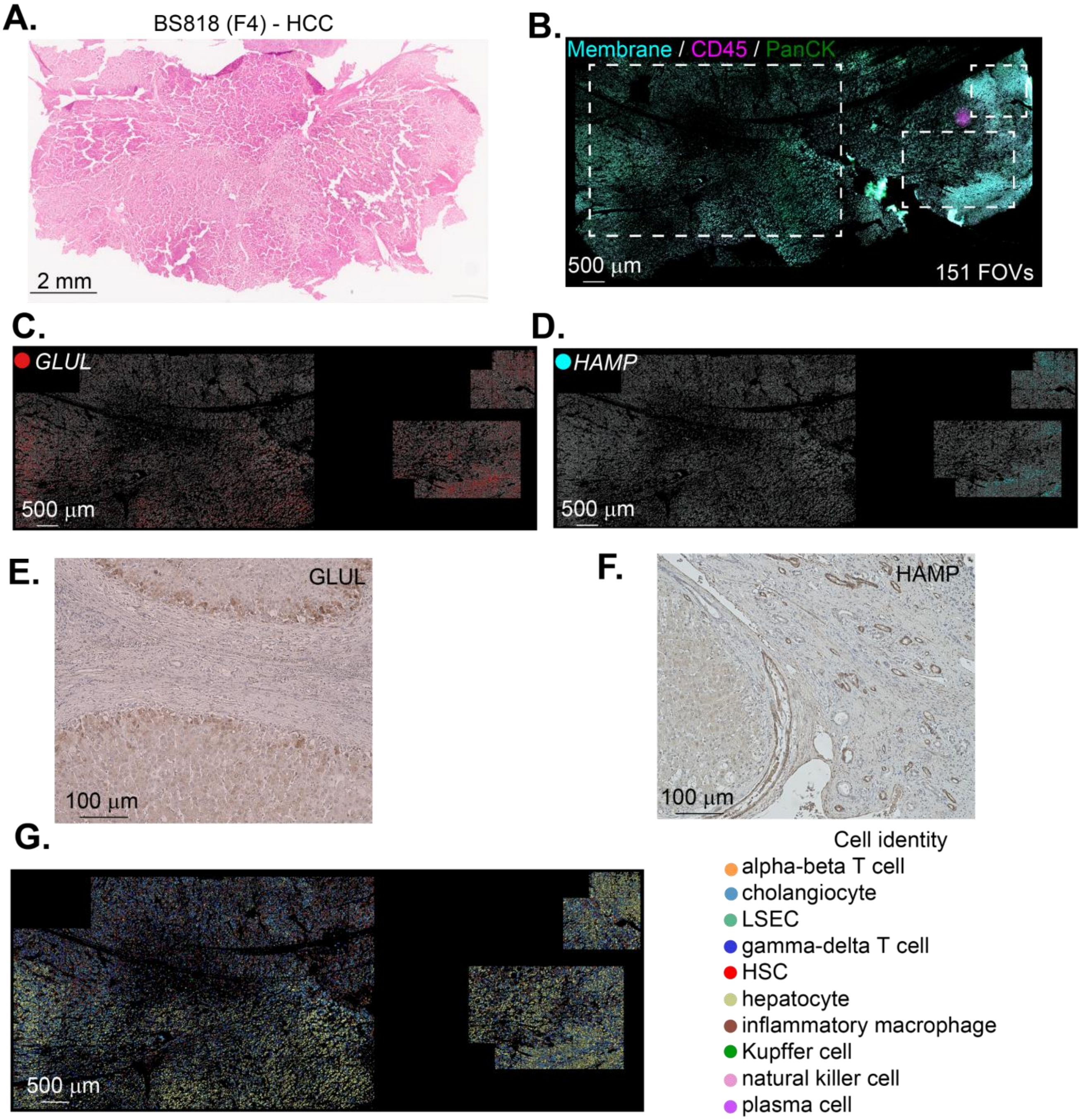

Figure S13: A) H&E, **B)** area sequenced by FOVs (n=151). **C)** *GLUL* and **D)** *HAMP* expression by CosMx and **E-F)** IHC demonstrate central vein hepatocyte zonation by GS in zone 3 and HAMP in zones 1-2 in BS818 HCC tissue. **G)** Cell-type referencing of BS818 – HCC.

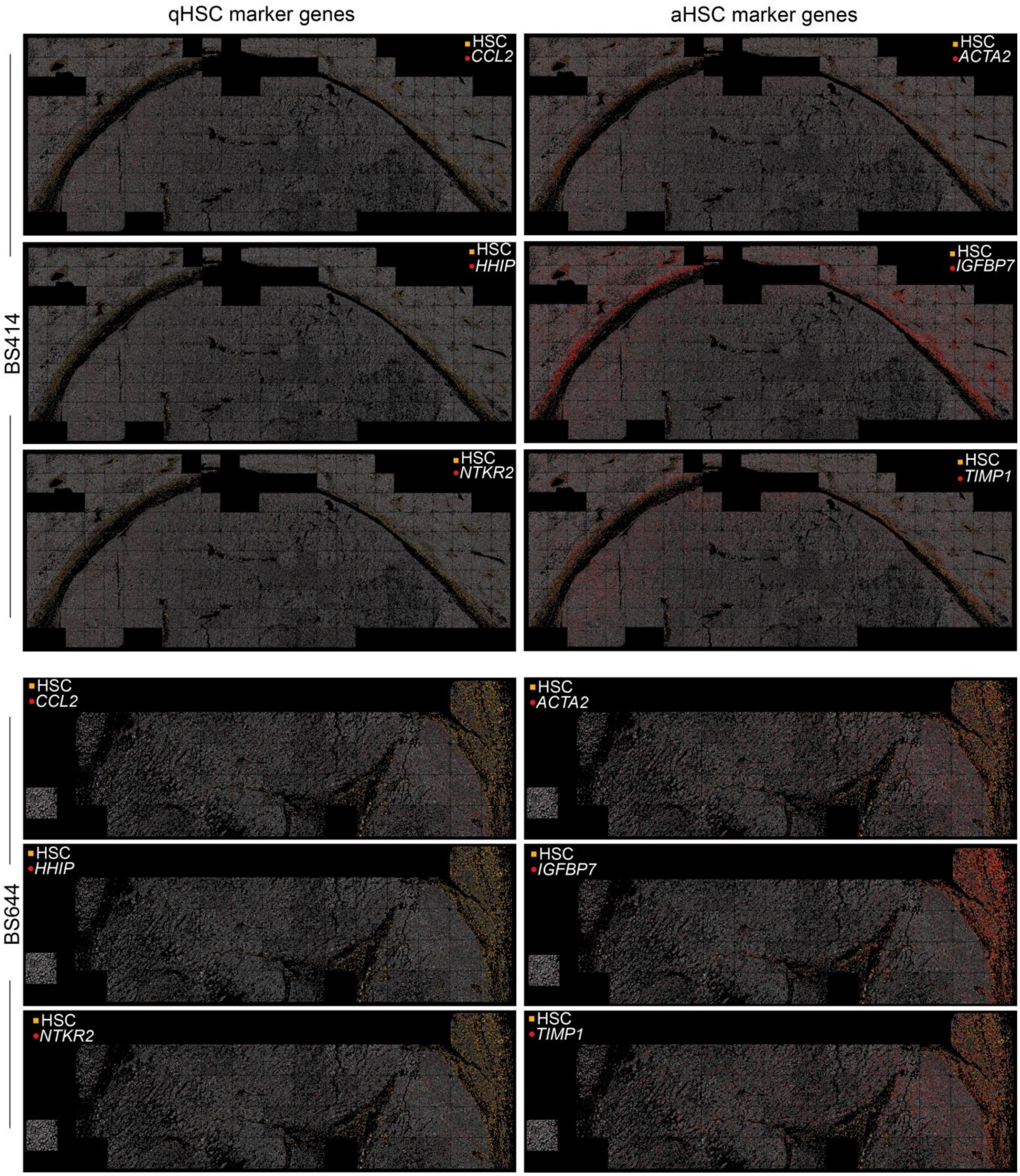

Figure S14: Spatial imaging of select quiescent and activated HSC genes (red) overlaid with HSCs populations (yellow) for HCC samples with pseudocapsules (BS414 and BS644). Activated HSC marker predominate in the pseudocapsule.

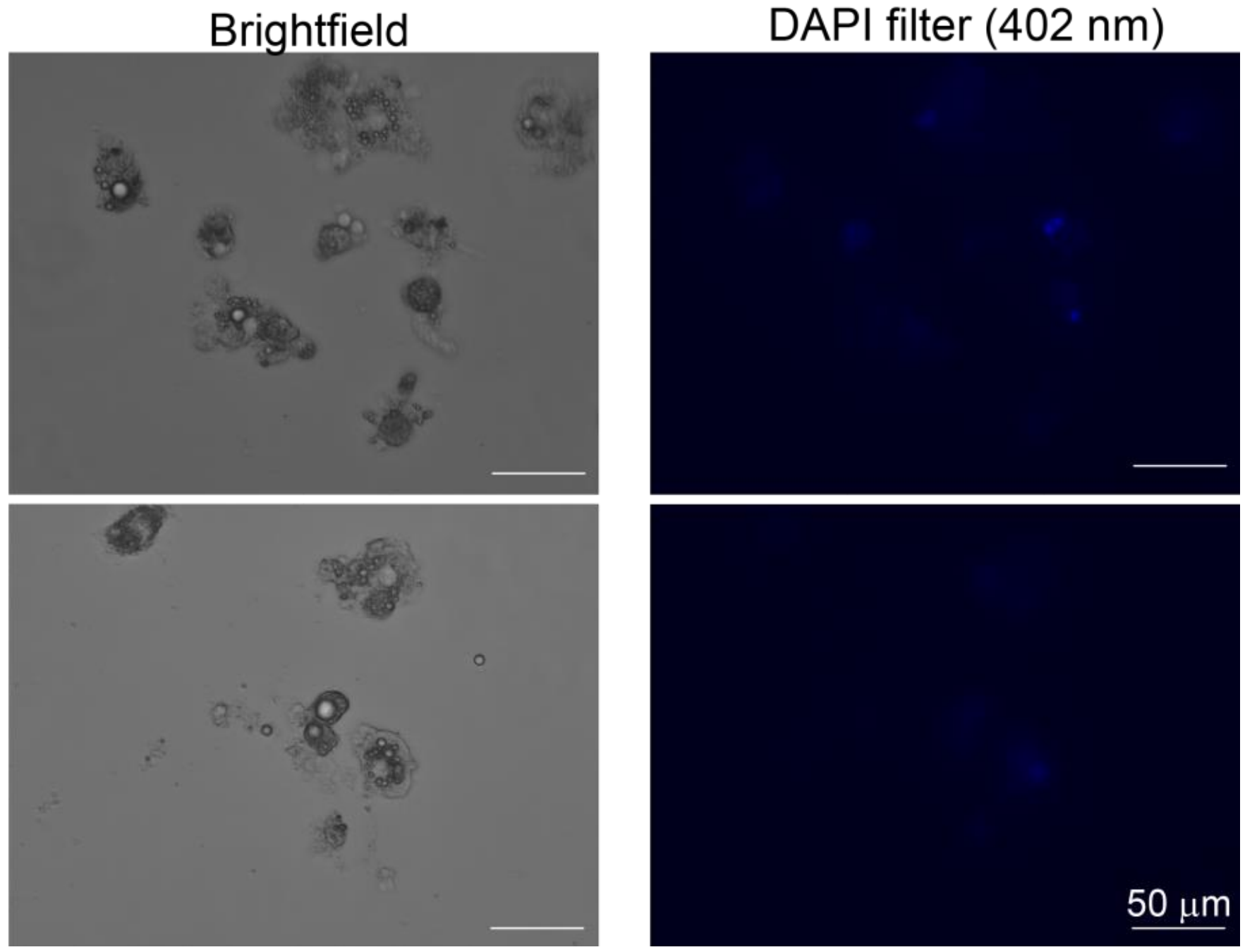

Figure S15: A) Quiescent HSCs have lipid droplets with autofluorescence due to vitamin A. Cells from donor #1 and were seeded and visualized day 2 post-seeding prior to cellular activation

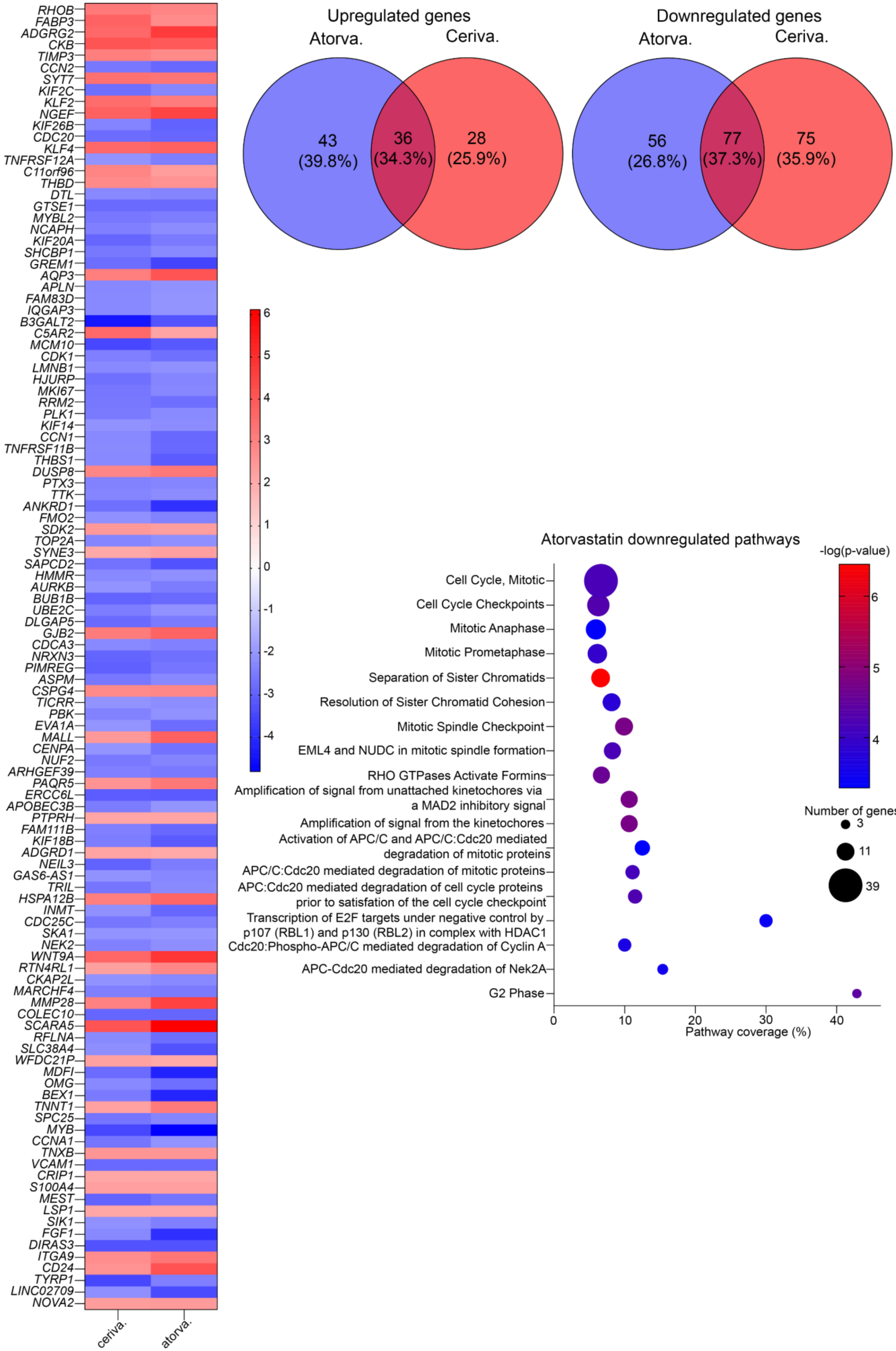
RHOB
FABP3
ADGRG2
CKB
TIMP3
CCN2
SYT7
KIF2C
KLF2
NGEF
KIF26B
CDC20
KLF4
TNFRSF12A
C11orf96
THBD
DTL
GTSE1
MYBL2
NCAPH
KIF20A
SHCBP1
GREM1
AQP3
APLN
FAM83D
IQGAP3
B3GALT2
C5AR2
MCM10
CDK1
LMNB1
HJURP
MKI67
RRM2
PLK1
KIF14
CCN1
TNFRSF11B
THBS1
DUSP8
PTX3
TTK
ANKRD1
FMO2
SDK2
TOP2A
SYNE3
SAPCD2
HMMR
AURKB
BUB1B
UBE2C
DLGAP5
GJB2
CDCA3
NRXN3
PIMREG
ASPM
CSPG4
TICRR
PBK
EVA1A
MALL
CENPA
NUF2
ARHGEF39
PAQR5
ERCC6L
APOBEC3B
PTPRH
FAM111B
KIF18B
ADGRD1
NEIL3
GAS6-AS1
TRIL
HSPA12B
INMT
CDC25C
SKA1
NEK2
WNT9A
RTN4RL1
CKAP2L
MARCHF4
MMP28
COLEC10
SCARA5
RFLNA
SLC38A4
WFDC21P
MDFI
OMG
BEX1
TNNT1
SPC25
MYB
CCNA1
TNXB
VCAM1
CRIP1
S100A4
MEST
LSP1
SIK1
FGF1
DIRAS3
ITGA9
CD24
TYRP1
LINC02709
NOVA2
ceriva.
atorva.
6
5
4
3
2
1
0
-1
-2
-3
-4
Upregulated genes
Atorva.
Ceriva.
43
(39.8%)
36
(34.3%)
28
(25.9%)
Downregulated genes
Atorva.
Ceriva.
56
(26.8%)
77
(37.3%)
75
(35.9%)
Atorvastatin downregulated pathways
Cell Cycle, Mitotic
Cell Cycle Checkpoints
Mitotic Anaphase
Mitotic Prometaphase
Separation of Sister Chromatids
Resolution of Sister Chromatid Cohesion
Mitotic Spindle Checkpoint
EML4 and NUDC in mitotic spindle formation
RHO GTPases Activate Formins
Amplification of signal from unattached kinetochores via a MAD2 inhibitory signal
Amplification of signal from the kinetochores
Activation of APC/C and APC/C:Cdc20 mediated degradation of mitotic proteins
APC/C:Cdc20 mediated degradation of mitotic proteins
APC:Cdc20 mediated degradation of cell cycle proteins prior to satisfaction of the cell cycle checkpoint
Transcription of E2F targets under negative control by p107 (RBL1) and p130 (RBL2) in complex with HDAC1
Cdc20:Phospho-APC/C mediated degradation of Cyclin A
APC-Cdc20 mediated degradation of Nek2A
G2 Phase
0
10
20
30
40
Pathway coverage (%)
-log(p-value)
6
5
4
Number of genes
3
11
39

Figure S16: Heatmap of all 113 common DEGs and Venn diagram of upregulated genes with cerivastatin (ceriva.) and atorvastatin (atorva.) treatments. Venn diagrams of the 113 DEGs separated into up- and downregulated by statin treatment. Downregulated Reactome pathways after atorvastatin treatment. There were no significant upregulated pathways with either statin treatment.

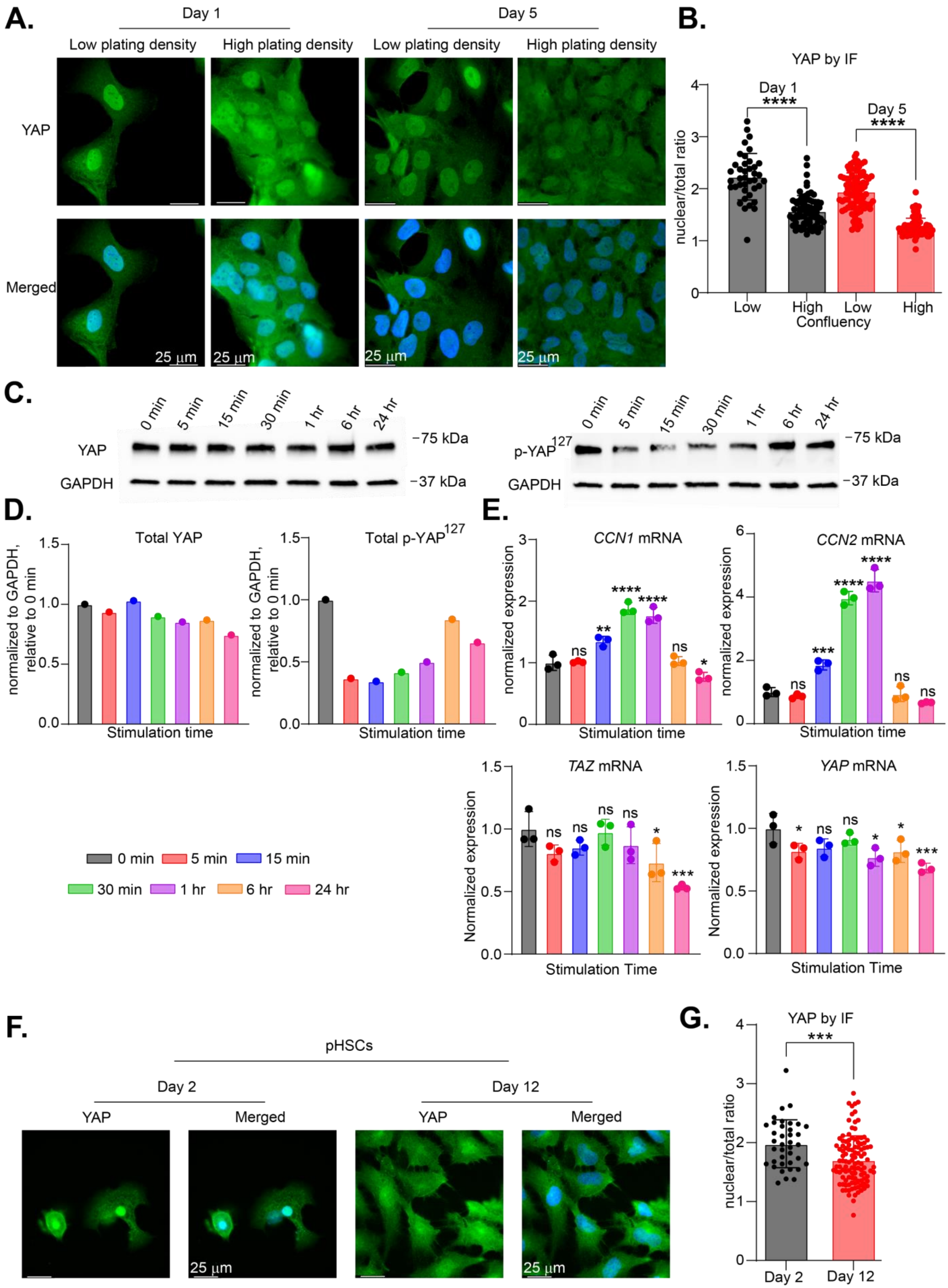
A.
Day 1
Day 5
Low plating density
High plating density
Low plating density
High plating density
YAP
Merged
25 μm
B.
YAP by IF
nuclear/total ratio
Day 1
****
Day 5
****
Low
High
Low
High
Confluency
C.
0 min
5 min
15 min
30 min
1 hr
6 hr
24 hr
YAP
GAPDH
75 kDa
37 kDa
p-YAP127
D.
Total YAP
Total p-YAP127
normalized to GAPDH, relative to 0 min
Stimulation time
E.
CCN1 mRNA
CCN2 mRNA
TAZ mRNA
YAP mRNA
normalized expression
Normalized expression
Stimulation time
Stimulation Time
ns
0 min
5 min
15 min
30 min
1 hr
6 hr
24 hr
F.
pHSCs
Day 2
Day 12
YAP
Merged
25 μm
G.
YAP by IF
***
nuclear/total ratio
Day 2
Day 12

Figure S17: A,B) YAP protein is predominantly localized to the nucleus at low cellular density compared to high density at both day 1 and 5 post plating by IF (*n*=39–73: day 1 and *n*=82-86: day 5, ****P<0.0001 by Mann–Whitney *U* test. **C,D)** Western blot analysis of both YAP and p-YAP^{127} after overnight serum starvation (0.5% FBS) and then re-stimulation with 10% FBS at different time points. **E)** mRNA levels of downstream YAP-TEAD target genes, *CCN1* and *CCN2*, increase with decreased p-YAP^{127} at 5min post re-stimulation confirming increased nuclear YAP, before returning to baseline at 6h but serum starvation does not significantly alter *TAZ* and *YAP* expression unless the stellate cells were refed for at least 6 hours with 10% FBS. Identifiers of significance are all relative to 0min of serum stimulation. *n*=3, *P < 0.0480, **P=0.0016, ***P < 0.0008, ****P<0.0001 by one-way ANOVA **F-G)** YAP was predominantly nuclear at low density (day 2 post seeding, quiescent) and cytosolic at high density (day 12, activated) in pHSCs. *n*=38–111, ***P=0.0004 by Mann–Whitney *U* test.

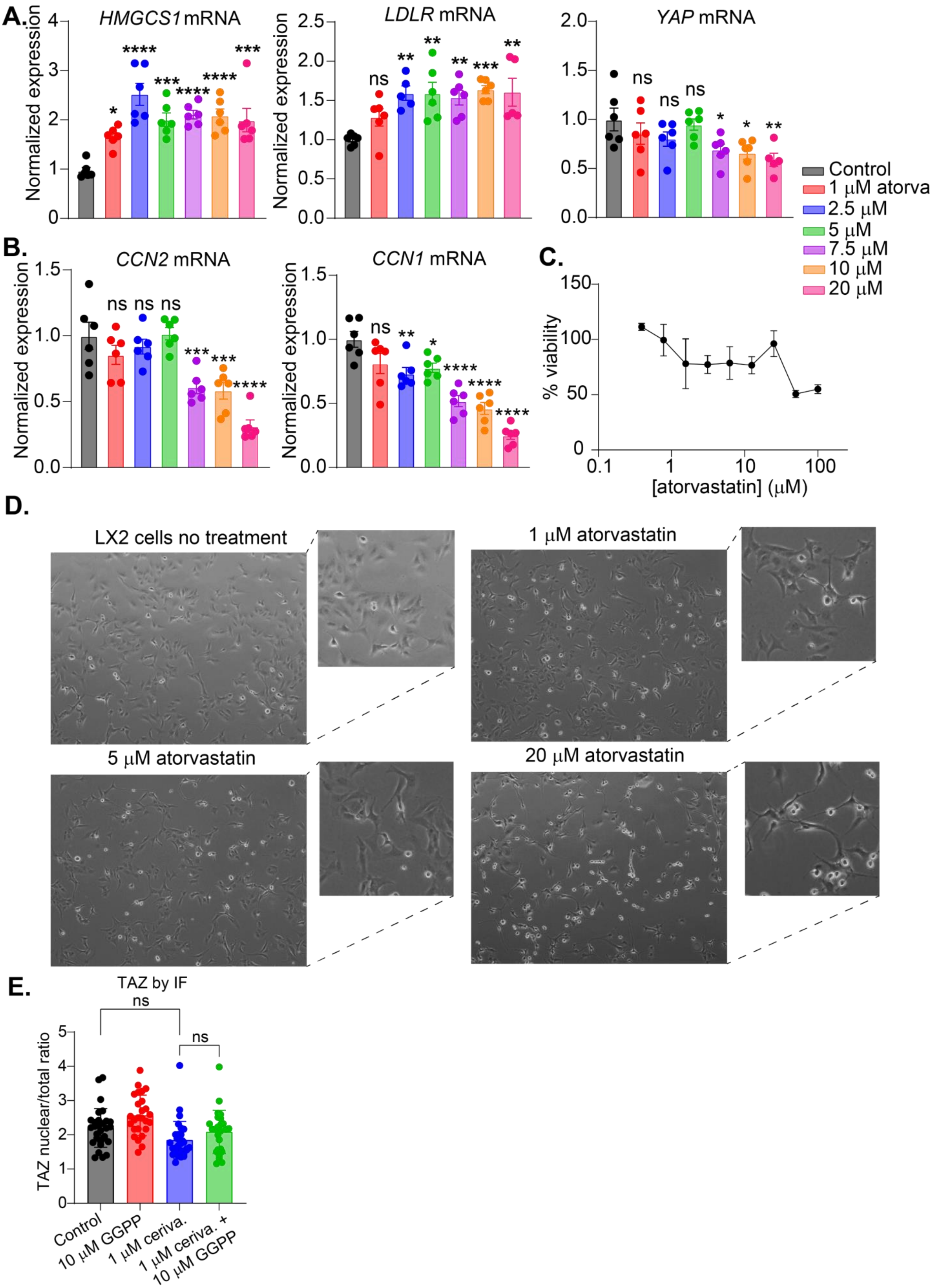
A.
HMGCS1 mRNA
LDLR mRNA
YAP mRNA
Normalized expression
Control
1 μM atorva
2.5 μM
5 μM
7.5 μM
10 μM
20 μM
B.
CCN2 mRNA
CCN1 mRNA
C.
% viability
[atorvastatin] (μM)
D.
LX2 cells no treatment
1 μM atorvastatin
5 μM atorvastatin
20 μM atorvastatin
E.
TAZ by IF
TAZ nuclear/total ratio
Control
10 μM GGPP
1 μM ceriva.
1 μM ceriva. + 10 μM GGPP

Figure S18: Atorvastatin dose responses for **A)** SREBP2 genes and **B)** YAP-TEAD genes. Identifiers of significance are all relative to control. *n* = 6, **P* < 0.0459, ***P* < 0.0078, ****P* < 0.0008, **P* < 0.0001, by one-way ANOVA. **C)** Cell viability of LX2 treated with 0 – 100 µM atorvastatin was assessed to ensure majority of LX2 cells were alive with the relatively high 20 µM dose of the lipophilic statin. *n* = 3. **D)** YAP-TEAD gene expression changes were not seen until high doses (7.5 µM and higher) of atorvastatin. This result can be visualized by cytoskeleton changes by brightfield images of the cells after 24 hours of treatment. **E)** TAZ nuclear/total ratio was quantified with cerivastatin and GGPP treatment. TAZ localization does not significantly change from nuclear to cytoplasmic with a 24 hour treat of cerivastatin and GGPP supplementation is unable to reverse the marginally effect. *n*= 25–30, *P* = *ns* by one-way ANOVA.

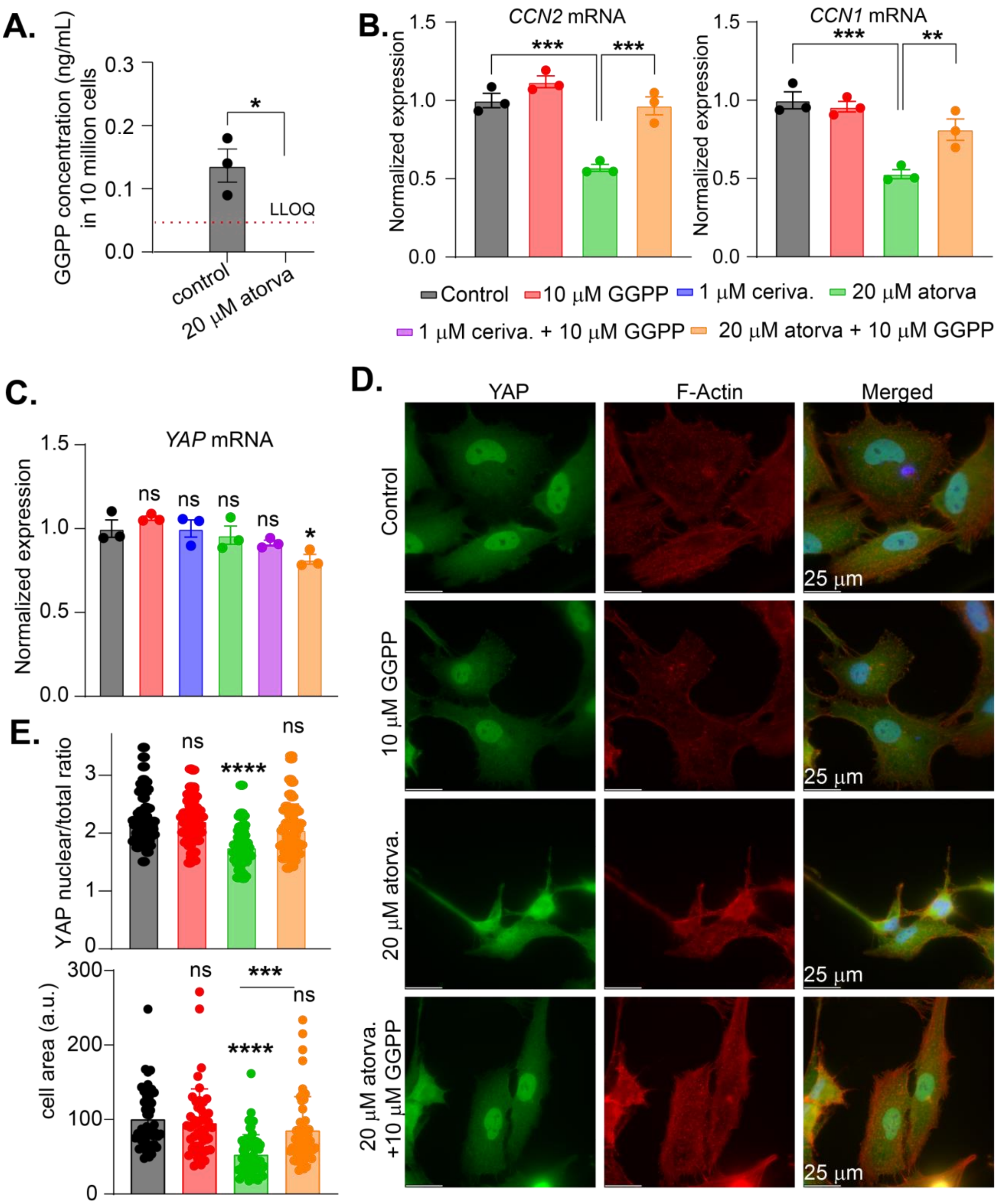

Figure S19: A) Atorvastatin was used to replicate the effects of cerivastatin seen in LX2 cells. Atorvastatin also altered YAP-TEAD genes *CCN1* and *CCN2* and GGPP supplementation recovered the YAP regulated gene expression. $n = 3$, not labeled, but there was no significant change between the control and 10 µM GGPP on the expression of *CCN1* and *CCN2*. Additionally, there was no significant difference for between the control

and atorvastatin with GGPP for either *CCN1* or *CCN2* expression. *YAP* expression is not significantly altered for any condition besides a slight difference between the control and atorva. + GGPP. **P* = 0.0274, ***P* = 0.0059, ****P* < 0.0003 by one-way ANOVA. **B)** YAP localization and cytoskeleton changes were visualized by IF and **C)** YAP nuclear/total ratio was quantified with atorvastatin and GGPP treatment. YAP localization changes from nuclear to cytoplasmic with a 24 hour treat of atorvastatin and GGPP supplementation is able to reverse the YAP cytoplasmic retention and the cytoskeleton shrinkage. *n* = 48–56, *****P* < 0.0001 by one-way ANOVA. Identifiers of significance are all relative to control. **D)** Measurement of cellular LX2 GGPP levels with and without 20μM atorvastatin by UPLC-MS/MS. Treatment reduced the level of GGPP below lower limit of quantification (LLOQ), but the change to the LLOQ was still significant. N=3 (**P*=0.0281 by Student's *t* test).

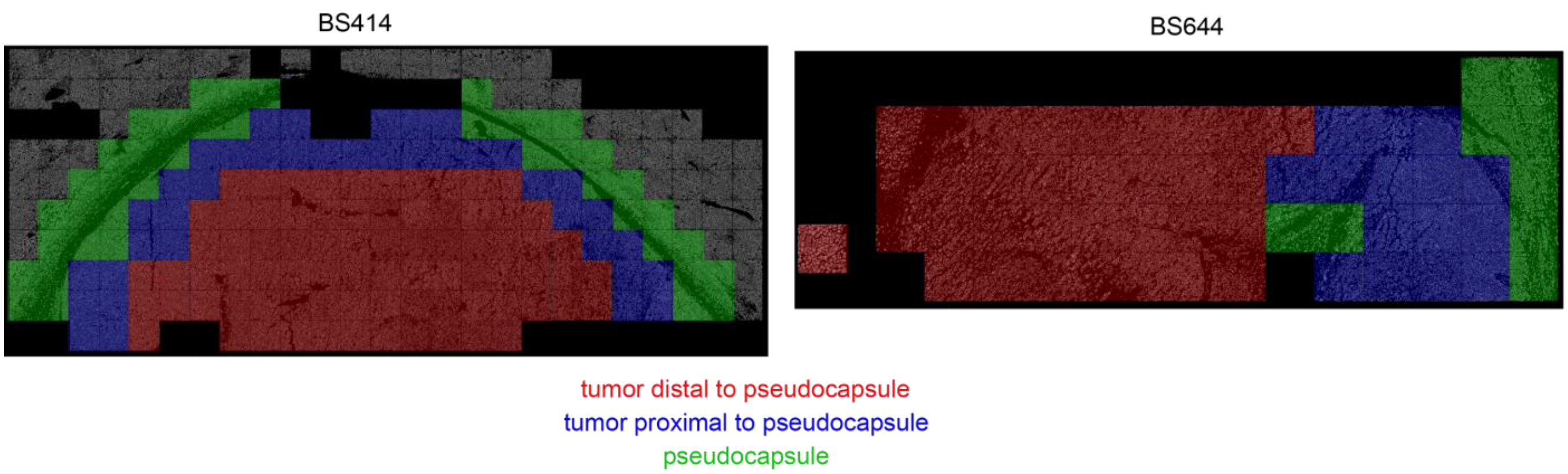

Figure S20: FOV maps of BS414 and BS644 following sectioning relative to the peritumoral pseudocapsule.

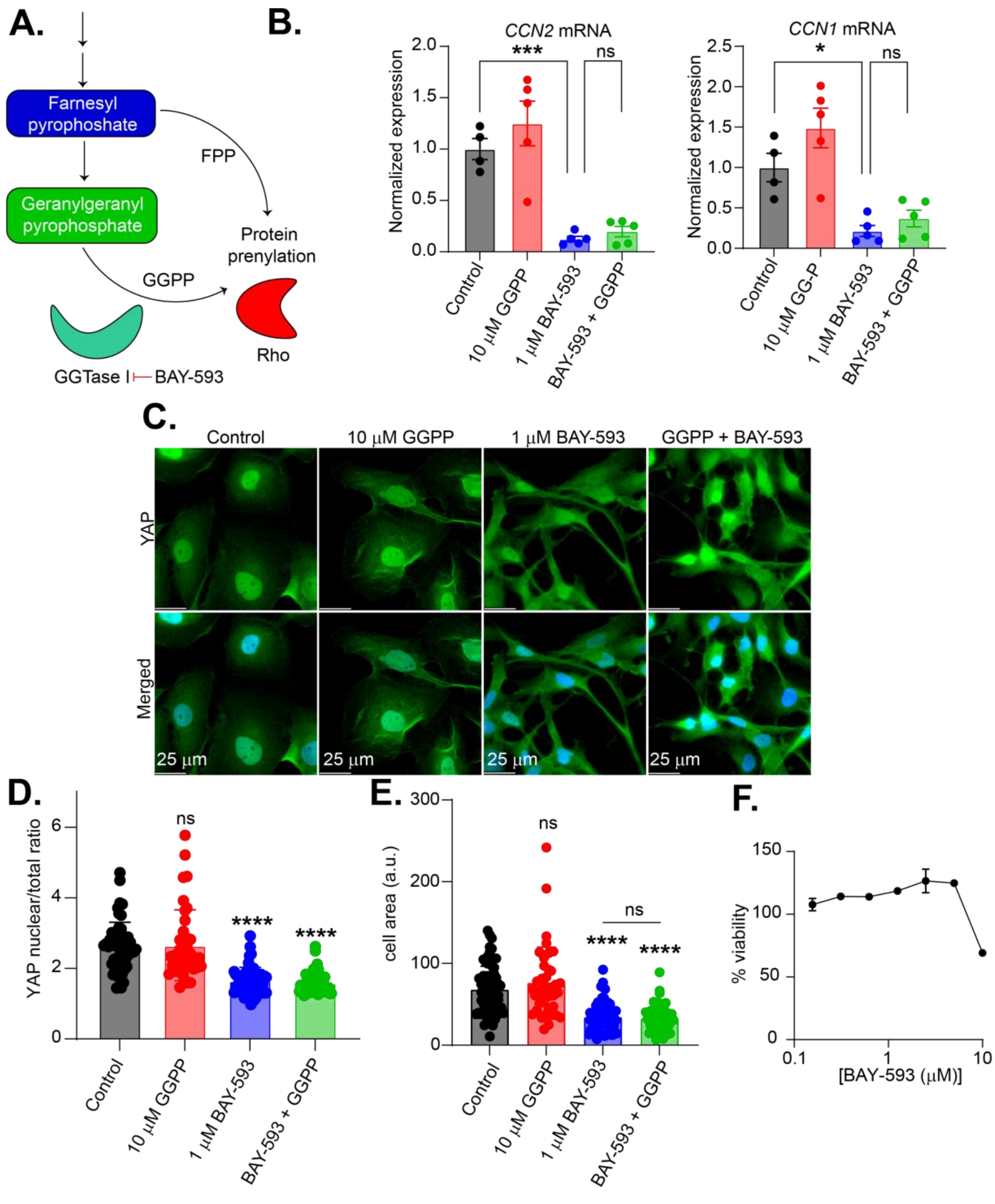

Figure S21: Effects of GGTase I inhibition with BAY-593. **A)** Schematic representation of BAY-593 mechanism of action**. B)** YAP-TEAD gene expression changes with 24 h BAY-593 treatment and GGPP repletion. *n* = 5. Not labeled, but there was no significant change between vehicle control and 10 µM GGPP. *P = 0.0124, ***P = 0.0007 by one-way ANOVA**. C)** YAP localization and cytoskeleton changes were visualized by IF and **D-E)** YAP nuclear/total ratio was quantified with BAY-593. YAP localization changes from nuclear to cytoplasmic with a 24-hour treatment of BAY-593 along with cytoskeleton shrinkage. *n* = 27-37, **P = 0.0048 by one-way ANOVA. **F)** Cell viability of LX2 treated with 0 – 10 µM BAY-593 was assessed to check for cytotoxicity. *n* = 3.

References

1. Rao S, Yang X, Ohshiro K, et al. β2-spectrin (SPTBN1) as a therapeutic target for diet-induced liver disease and preventing cancer development. Science Translational Medicine 2021;13:eabk2267.
2. Alvarez M, Benhammou JN, Rao S, et al. Isolation of nuclei from human snap-frozen liver tissue for single-nucleus RNA sequencing. Bio-protocol 2023;13:e4601.
3. Kaminow B, Yunusov D, Dobin A. STARsolo: accurate, fast and versatile mapping/quantification of single-cell and single-nucleus RNA-seq data. Biorxiv 2021:2021.05. 05.442755.
4. Fleming SJ, Chaffin MD, Arduini A, et al. Unsupervised removal of systematic background noise from droplet-based single-cell experiments using CellBender. Nature methods 2023;20:1323–1335.
5. Neavin D, Senabouth A, Arora H, et al. Demuxafy: improvement in droplet assignment by integrating multiple single-cell demultiplexing and doublet detection methods. Genome biology 2024;25:94.
6. Wolf FA, Angerer P, Theis FJ. SCANPY: large-scale single-cell gene expression data analysis. Genome biology 2018;19:15.
7. Andrews TS, Atif J, Liu JC, et al. Single-Cell, Single-Nucleus, and Spatial RNA Sequencing of the Human Liver Identifies Cholangiocyte and Mesenchymal Heterogeneity. Hepatol Commun 2022;6:821–840.
8. MacParland SA, Liu JC, Ma XZ, et al. Single cell RNA sequencing of human liver reveals distinct intrahepatic macrophage populations. Nat Commun 2018;9:4383.
9. Guilliams M, Bonnardel J, Haest B, et al. Spatial proteogenomics reveals distinct and evolutionarily conserved hepatic macrophage niches. Cell 2022;185:379–396 e38.
10. Xu C, Prete M, Webb S, et al. Automatic cell-type harmonization and integration across Human Cell Atlas datasets. Cell 2023;186:5876–5891. e20.
11. Domínguez Conde C, Xu C, Jarvis LB, et al. Cross-tissue immune cell analysis reveals tissue-specific features in humans. Science 2022;376:eabl5197.
12. Alvarez M, Benhammou JN, Darci-Maher N, et al. Human liver single nucleus and single cell RNA sequencing identify a hepatocellular carcinoma-associated cell-type affecting survival. Genome medicine 2022;14:50–50.
13. Kim MC, Gate R, Lee DS, et al. Method of moments framework for differential expression analysis of single-cell RNA sequencing data. Cell 2024;187:6393–6410. e16.
14. Traag VA, Waltman L, Van Eck NJ. From Louvain to Leiden: guaranteeing well-connected communities. Scientific reports 2019;9:1–12.
15. Cogliati B, Yashaswini CN, Wang S, et al. Friend or foe? The elusive role of hepatic stellate cells in liver cancer. Nat Rev Gastroenterol Hepatol 2023.
16. He S, Bhatt R, Brown C, et al. High-plex imaging of RNA and proteins at subcellular resolution in fixed tissue by spatial molecular imaging. Nature biotechnology 2022;40:1794–1806.
17. Pachitariu M, Rariden M, Stringer C. Cellpose-SAM: superhuman generalization for cellular segmentation. bioRxiv 2025:2025.04. 28.651001.
18. Hao Y, Stuart T, Kowalski MH, et al. Dictionary learning for integrative, multimodal and scalable single-cell analysis. Nature biotechnology 2024;42:293–304.
19. Danaher P, Zhao E, Yang Z, et al. Insitutype: likelihood-based cell typing for single cell spatial transcriptomics. BioRxiv 2022:2022.10. 19.512902.
20. Vasimuddin M, Misra S, Li H, et al. Efficient architecture-aware acceleration of BWA-MEM for multicore systems, In 2019 IEEE international parallel and distributed processing symposium (IPDPS), IEEE, 2019.
21. Regier AA, Farjoun Y, Larson DE, et al. Functional equivalence of genome sequencing analysis pipelines enables harmonized variant calling across human genetics projects. Nature communications 2018;9:4038.
22. Talevich E, Shain AH, Botton T, et al. CNVkit: genome-wide copy number detection and visualization from targeted DNA sequencing. PLoS computational biology 2016;12:e1004873.
23. Fabregat A, Jupe S, Matthews L, et al. The reactome pathway knowledgebase. Nucleic acids research 2018;46:D649–D655.

24. Fabregat A, Sidiropoulos K, Viteri G, et al. Reactome pathway analysis: a high-performance in-memory approach. BMC bioinformatics 2017;18:1–9.
25. Bhattacharya D, Basta B, Mato JM, et al. Aramchol downregulates stearoyl CoA-desaturase 1 in hepatic stellate cells to attenuate cellular fibrogenesis. JHEP Rep 2021;3:100237.
26. Frankish A, Diekhans M, Ferreira A-M, et al. GENCODE reference annotation for the human and mouse genomes. Nucleic acids research 2019;47:D766–D773.
27. Chen S, Zhou Y, Chen Y, et al. fastp: an ultra-fast all-in-one FASTQ preprocessor. Bioinformatics 2018;34:i884–i890.
28. Ewels P, Magnusson M, Lundin S, et al. MultiQC: summarize analysis results for multiple tools and samples in a single report. Bioinformatics 2016;32:3047–3048.
29. Bray NL, Pimentel H, Melsted P, et al. Near-optimal probabilistic RNA-seq quantification. Nature biotechnology 2016;34:525–527.
30. Soneson C, Love MI, Robinson MD. Differential analyses for RNA-seq: transcript-level estimates improve gene-level inferences. F1000Research 2015;4.
31. Love MI, Huber W, Anders S. Moderated estimation of fold change and dispersion for RNA-seq data with DESeq2. Genome biology 2014;15:1–21.
32. Slowikowski K, Schep A, Hughes S, et al. Package ggrepel. Automatically position non-overlapping text labels with ‘ggplot2 2018.
33. Wickham H, Wickham H. Programming with ggplot2. Ggplot2: elegant graphics for data analysis 2016:241–253.
